# Supplementary material for: TRPM7 deficiency exacerbates cardiovascular and renal damage induced by aldosterone-salt
Source: Commun Biol. 2022 Jul 26;5:746. doi: 10.1038/s42003-022-03715-z (PMC9325869; doi:10.1038/s42003-022-03715-z)

SUPPLEMENTARY TEXT

TRPM7 deficiency exacerbates cardiovascular and renal damage induced by aldosterone-salt

*Francisco J Rios[1], Zhi-Guo Zou[1], Adam P. Harvey[1], Katie Y. Harvey[1], Livia L. Camargo[1], Karla B Neves[1], Sarah E.F. Nichol[1], Rheure A. Lopes[1], Alexius Cheah[1], Maram Zahraa[1], Alexey G Ryazanov[2], Lillia Ryazanova[3], Thomas Gudermann[4], Vladimir Chubanov[4], Augusto C. Montezano[1], *Rhian M. Touyz[1 5].

[1] Institute of Cardiovascular and Medical Sciences, BHF Glasgow Cardiovascular Research Centre, University of Glasgow, Glasgow, United Kingdom.
[2] Department of Pharmacology, Rutgers Robert Wood Johnson Medical School, USA. [3]Lewis Sigler Institute of Integrative Genomics, Princeton University, Princeton, USA. [4]Walther-Straub Institute of Pharmacology and Toxicology, Ludwig-Maximilians-Universität München, Munich, Germany. [5]Research Institute of McGill University Health Centre, McGill University, Montreal, Canada.

Running title: TRPM7 in cardiovascular damage

Keywords: TRPM7, magnesium, cardiac hypertrophy, vascular inflammation

*** Corresponding authors:**

Rhian M Touyz MBBCh, PhD

ORCID ID : https://orcid.org/0000-0003-0670-0887

1001, boul Décarie, ES1.5066.6

Montréal, Québec, Canada, H4A 3J1

tél./tel. (514) 934-1934 #71608

Email: rhian.touyz@mcgill.ca

Francisco J Rios PhD;
ORCID ID: https://orcid.org/0000-0002-8194-0787
Institute of Cardiovascular & Medical Sciences
BHF Glasgow Cardiovascular Research Centre
University of Glasgow
126 University Place, Glasgow G12 8TA
Tel: + 44 (0)141 330 7775/7774; Fax: + 44 (0)141 330-3360
Email. Francisco.Rios@glasgow.ac.uk

Supplementary Methods

Animals

Wild type (WT) mice (C57BL/6J and SV129 mixed background) and mice heterozygous for the deletion of the TRPM7-kinase (TRPM7$^{+/\Delta kinase}$), generated by the gene-targeting vector technique[1]. Briefly, the mutation was generated by inserting a Neo gene cassette between exons 32-36 of the kinase. As a result, the TRPM7 protein is truncated immediately upstream of the alpha-kinase domain. Using reverse transcription–PCR analysis we identified wildtype (WT) (TRPM7+/+) and heterozygous (TRPM7$^{+/\Delta kinase}$ (TRPM7+/Δ)) animals. Homozygous mice TRPM7$^{\Delta kinase/\Delta kinase}$ are embryonic lethal. Littermate mice were generated on a mixed C57Bl/6 and SV129 background. To generate WT and TRPM7$^{+/\Delta kinase}$, breeding pairs were formed with male WT and female TRPM7$^{+/\Delta kinase}$. The offspring can be either homozygous TRPM7+/+ (WT) or heterozygous (TRPM7$^{+/\Delta kinase}$), which are determined by genotyping at 3 weeks of age[1,2]. Mice were maintained in controlled room temperature (25°C) with a 12 h light/dark cycle and food and water *ad libitum.* Mice were fed standard chow. Genotyping was performed using the REDExtract-N-AmpTM Tissue PCR Kit (Sigma-Aldrich, Dorset, UK) according to manufacturer's instructions. Primers to detect the TRPM7$^{+/\Delta\ kinase}$ (5' tgc gag gcc aga ggc cac ttg tgt agc 3'; and 5' tgc gag gcc aga ggc cac ttg tgt agc 3') were designed to amplify the neighboring sequence regions of the TRPM7 kinase domain.

TRPM7 kinase-dead mice (TRPM7$^{R/R}$) mice has been reported previously [3] [4]. The TRPM7 locus contains a 'kinase-dead' K1646R point mutation, which specifically abrogates

the catalytic activity of TRPM7 kinase domain in the whole organism. Tissues from TRPM7 "kinase-dead" mice (TRPM7$^{R/R}$) and wild-type C57Bl/6 were provided by Dr. Chubanov, Ludwig-Maximilians Universität München. TRPM7$^{R/R}$ mice were handled according to the European Union Animal Welfare Act and the local councils on animal care (permits AZ: 55.1-8791-14.718 and 55.2-1-54-2532-180-2016 from Government of Oberbayern).

Animal treatment

WT and TRPM7$^{+/\Delta kinase}$ male mice, 12-16 weeks of age were studied. Surgical procedures were performed under anesthesia: 5% isoflurane in 1.5 L/min O_2, then reduced to 2.5% isoflurane in 1.5 L/min O_2 for maintenance. WT and TRPM7$^{+/\Delta kinase}$ mice were divided into 4 groups and treated for 4 weeks as follow: Group 1 - vehicle controls (veh, WT n=14; TRPM7$^{+/\Delta kinase}$ n= 17); Group 2 - aldosterone-infused (aldosterone group, WT n=10; TRPM7$^{+/\Delta kinase}$ n= 9) (600 µg/kg/day) (Sigma-Aldrich, Dorset, UK) by Alzet osmotic mini-pumps (model 2004, Alzet, CA, USA); Group 3 - 1% NaCl drinking water (salt group, WT n=7; TRPM7$^{+/\Delta kinase}$ n= 9); Group 4 - aldosterone-infused + 1% NaCl drinking water group (aldosterone-salt, WT n=13; TRPM7$^{+/\Delta kinase}$ n= 15). Mice were euthanized after 4 weeks of treatment by overdose of anesthesia, followed by cardiac puncture. Aorta, mesenteric vascular bed, heart, kidneys and spleens were dissected for further studies.

Plasma and urine biochemistry

Blood was collected under isoflurane anesthesia (5% in 1.5 L/min O2) by cardiac puncture immediately prior to sacrifice. Blood was collected in heparinized tubes (TekLab, County Durham, UK). Plasma was separated by centrifugation (2,000 rpm, 10 min) (Heraeus Megafuge 16R; ThermoScientific). Spot urine was collected from the bladder during sacrifice and snap frozen in liquid nitrogen. Plasma and urine were stored at -80 ^{o}C. Concentrations of calcium, phosphate, sodium, potassium, chloride, magnesium, albumin, creatinine, plasma glucose were determined by an automated analyzer (Roche/Hitachi cobas c systems - cobas c 311 Autoanalyser).

Culture of cardiac fibroblasts

Hearts from WT and TRPM7$^{+/\Delta kinase}$ mice were cut into small pieces and placed in digestion solution: ADS buffer (NaCl 116.4 mmol/L; KCl 5.4 mmol/L; glucose 5.6 mmol/L; NaH_2PO4 1.0 mmol/L; $MgSO_4$ 0.8 mmol/L; HEPES 20 mmol/L, pH 7.3) supplemented with collagenase 0.04% and pancreatin 0.03% (Sigma-Aldrich, Dorset, UK). The digestion process comprises 10 steps, all at 37 ^{o}C: 1) 10 min, 10 mL digestion solution, agitation speed 180 (strokes/min); 2) 10 min, 10 mL digestion solution, 160 strokes/min; 3-4) 8 min, 10 mL digestion solution,

150 strokes/min; 5) 6 min, 10 mL digestion solution, 160 strokes/min; 6-9) 6 min, 10 mL digestion solution, 150 strokes/min; 10) 30 min, 10 mL digestion solution, 150 strokes/min. After each digestion step, enzymes were inactivated with 2 ml FBS and centrifuged (5 min, 300 x g). Pellet was resuspended in 10 ml DMEM/F12 (10% FBS) and plated in T25 flask. Non-adherent cells were removed after 3 h and medium changed to DMEM/20% FBS. Fibroblasts were kept in culture and experiments were performed until passage 8. Part of the TRPM7$^{+/\Delta kinase}$ cells were constantly cultured in the presence of $MgCl_2$ (10 mM in DMEM/20% FBS). Fibroblasts were maintained in 0.5% FBS for 24 h before experiments. Cells were treated with aldosterone (10^{-7} mol/L) (Sigma-Aldrich, Dorset, UK) for short time simulation (10 min) or long time stimulation (4 and 24 h) and investigated for protein expression by immunoblotting.

Histology

Hearts, aortas, and kidneys were fixed in 10% buffered-formalin solution and processed for histological inclusion in paraffin. Five-µm thick tissue sections were stained with PicroSirius red for light microscopy. Fifteen randomly selected non-overlapping fields (200×) were analyzed using the package ImageJ 1.44p (Wayne Rasband, NIH, USA) available in http://imagej.nih.gov/ij.

Functional studies in mesenteric resistance arteries

First- and second-order mesenteric resistance arteries were cut into 2 mm ring segments and mounted on a wire myograph as previously described [5]. Myograph chambers were filled with 5 mL of Krebs–Henseleit physiological solution [(in mmol/L): NaCl 130, $NaHCO_3$ 14.9, KCl 4.7, KH_2PO_4 1.18, $MgSO_4$•7H_2O 1.17, glucose 5.5, $CaCl_2$•2H_2O 1.56, and EDTA 0.026] (all from Sigma-Aldrich, Dorset, UK) and continuously gassed with a mixture of 95% O_2 and 5% CO_2 at a temperature of 37º C. After 30 min of stabilization, contractile responses were assessed by adding KCl 120 mmol/L (Sigma-Aldrich, Dorset, UK) to the organ baths. Endothelial integrity was verified by relaxation induced by acetylcholine (Ach; 10^{-6} mol/L) (Sigma-Aldrich, Dorset, UK) in vessels pre-contracted with phenylephrine (Phe; 2×10^{-6} mol/L) (Sigma-Aldrich, Dorset, UK). Cumulative concentration–response curves to Phe (10^{-9} mol/L to 3×10^{-4} mol/L). Endothelium-dependent relaxation was assessed by concentration-responses to Ach 10^{-9} mol/L to 10^{-4} mol/L) in Phe-pre-contracted vessels. Endothelium-independent relaxation was assessed by concentration responses to sodium nitroprusside (SNP, 10^{-10} mol/L to 10^{-4} mol/L) (Sigma-Aldrich, Dorset, UK) in Phe-pre-contracted vessels.

Pressure myography

Vascular structure and mechanics were assessed in resistance arteries prepared as pressurized systems (Danish Myo Tech, Model 110P and 111P) as previously described [5]. Briefly, mesenteric small arteries were dissected and vessel segments were placed between two-glass microcannula in calcium-free Krebs solution containing EGTA 10 mmol/L and allowed to equilibrate for 30 min at 37°C, 95% O_2 and 5% CO_2. Pressure–diameter curve obtained by progressively increasing intraluminal pressure between 3 and 120 mmHg. Internal and external diameters were used to calculate parameters such as: wall thickness, cross-sectional area (CSA), and wall:lumen ratio [6]. Mechanical properties were assessed by stress-strain curves as previously described [5 6].

Measurement of tissue Mg^{2+}

Dried tissues were weighed using an analytical balance, followed by digestion in nitric acid concentrated in a water bath at 65 °C for 2 h with the lids closed. Samples were cleared by centrifugation at 10,000 x g for 1 min, diluted 1/10 in milliQ H_2O and further incubated in a water bath 65 °C for 2 h with the lids closed. Samples were centrifuged at 10,000 x g for 1 min. 5 µL of digested samples were transferred to microplates and the Mg^{2+} concentration was analyzed by colorimetric reaction using the commercial kit Magnesium Gen.2 (Roche Diagnostics, Burgess Hill, UK). Absorbance at 600 nm was measured using a microplate reader Spectramax M (Molecular devices, Winnersh, UK). Concentration in mmol/L was obtained using a standard curve prepared with $MgCl_2$ and normalized by dried weigh, as described [7].

Sample preparation for Flow Cytometry

Kidneys were cut in small pieces ≤ 1 mm and digested in collagenase II (2 mg/mL) (Sigma-Aldrich, Dorset, UK) diluted in DMEM serum free with constant agitation at 37 ºC for 40 min. Enzyme was inactivated by adding FBS 10% in final concentration. Digested tissues were filtered through a 70 µm cell strainer. Spleen cells were collected after mechanic disruption and filtered through a 70 µm cell strainer. Cell suspension obtained from kidneys and spleens were centrifuged at 300 x g for 10 min. The cell pellet was resuspended in ACK Lysis Buffer (NH_4Cl 150 mmol/L, $KHCO_3$ 10 mmol/L, Na_2EDTA 0.1 mmol/L, pH 7.2–7.4) for 3 min on ice for erythrocytes disruption, followed by addition of 40 mL of PBS/FBS 2% and centrifugation at 300 x g for 10 min. Total cell number was obtained by counting in a Neubauer chamber, using trypan blue 0.4% exclusion.

Flow Cytometry

Cells from kidneys and spleens were resuspended in FACS buffer (PBS/FBS 2%, NaN_3 0.05%). Unspecific biding was blocked by 5% normal rat serum in FACS buffer for 15 min on ice. Cells were washed, resuspend in FACS buffer and stained with fluorescent-conjugated

anti-mouse monoclonal antibodies for 30 min on ice and in the dark: anti-CD45-FITC (30-F11), anti-CD3-PE-Cy7 (145-2C11), anti-CD4-APC (GK1.5), anti-CD8-APC-Cy7 (53-6.7), anti-F4/80-Alexa-647 (BM8), anti-CD11c-PE-Cy7 (N418), anti-CD206-FITC (C068C2) all from Biolegend (London, UK) and anti-CD45-PE (30-F11, BD Pharmingen, Oxford, UK). Cells were washed twice in PBS and resuspended in 200 µL of FACS buffer. Data acquisition was performed in a FACS Canto II flow cytometer (BD Biosciences, Wokingham, UK) and analyzed using FlowJo software (TreeStar, Ashland, USA).

Real-Time Reverse-Transcription Polymerase Chain Reaction (PCR).

Total RNA was isolated using the QIAzol Lysis Reagent (Qiagen, Manchester, UK) according to the manufacturer's instructions and diluted in nuclease-free H_2O (Ambion/Life Technologies, Paisley, UK). cDNA was generated from total RNA using the High-Capacity cDNA Reverse Transcription Kits (Applied Biosystems, Warrington, UK). Real-time polymerase chain reaction was performed with the Applied Biosystems QuantStudio 12K Flex real time PCR system, using Power SyBr Green Master Mix (Applied Biosystems, Warrington, UK) and specific murine primers to *GAPDH*, *TRPM7*, *TRPM6*, fibronectin (*FN1*) and collagen-1 (*Col1a1*). All acquired from Eurofins genomics (Glasgow, UK) (**Supplemental Table 1**). Relative gene expression was calculated by the $2^{-\Delta\Delta Ct}$ cycle threshold method as previously described [8].

Sample preparation for Immunoblotting

Total protein from frozen tissues and cells was extracted in lysis buffer containing Tris 50 mmol/L, pH 8.0, NaCl 150 mmol/L, Triton X-100 1%, SDS 0.1%, supplemented with phenylmethylsulfonyl fluoride (PMSF) 1 mmol/L, pepstatin A 1 µg/mL, leupeptin 1 µg/mL, aprotinin 1 µg/mL (Sigma-Aldrich, Dorset, UK), sodium fluorate 10 mmol/L (AnalaR Normapur; VWR International, Leuven, Belgium), and sodium orthovanadate 1 mmol/L (Alfa Aesar, Heysham, UK). Total protein lysate was sonicated twice (10 kHz, 5 sec, 4ºC), cleared by centrifugation at 10,000 rpm for 5 min and the pellet was discarded. Protein concentration was determined using the DC protein assay kit (Pierce – ThermoFisher Scientific).

Immunoblotting

Proteins from kidneys and spleens (30 µg) were separated by electrophoresis on a polyacrylamide gel and transferred onto a nitrocellulose membrane (Thermo Scientific, Darmstadt, Germany). Nonspecific binding sites were blocked with 5% non-fatty dry milk solubilized in Tris-buffered saline solution with Tween 0.01 % for 1 hour at room temperature. Membranes were then incubated overnight at 4°C with the following primary specific

antibodies: β-actin (mouse), Nox1 (goat) (Sigma-Aldrich, Dorset, UK); phospho-Smad3 (mouse, S423 + S425), peroxiredoxin-SO3 (Prs-SO_3H, rabbit), α-tubulin (rabbit), IL-6 (rabbit), TGFβ1 (rabbit) (Abcam, Cambridge, UK); phospho-Stat3 (Tyr705) (mouse), total-Stat3 (rabbit), phospho-Stat1 (Tyr701) (rabbit), total-Stat1 (mouse), phospho-ERK1/2 (Thr202/Tyr204, rabbit), total-Smad3 (rabbit) (Cell Signaling Technology, Beverly, MA, USA); total-ERK1/2 (mouse), PTEN (mouse) (Santa Cruz Biotechnology, Heidelberg, Germany, Inc); IL-11 (mouse) (Biolegend London, UK), ox-PTP (mouse), PPM1A (mouse) (R&D Systems, Abingdon, UK) phospho-TRPM7 (Ser1511, rabbit)[9]; Nox4, Nox2, αENaC (rabbit, Abcam, Cambridge, UK); Na+/K+ ATPase 1 (ATP1A1, rabbit, Proteintech Europe, Machester, UK) Next, membranes were washed with TBS-tween and incubated with secondary fluorescence-coupled antibodies goat-anti-mouse-IRDye 680 or goat-anti-rabbit-IRDye 800 (LI-COR, Cambridge, UK) 1 h, at room temperature in the dark and visualized by an infrared laser scanner (Odyssey Clx, LICOR, Cambridge, UK). Images were quantified using the software Image Studio™ Lite free version (LICOR, Cambridge, UK). Protein expression levels were normalized to loading controls.

Proliferation assay

The cell tracking dye carboxyfluorescein succinimidyl ester (CFSE) (Sigma-Aldrich, Dorset, UK) was used to assess cardiac fibroblast proliferation. CSFE is a fluorescent cell staining dye that can be used to monitor cell proliferation due to the progressive halving of CFSE fluorescence within daughter cells following each cell division. Therefore, reduced fluorescence is associated with cell proliferation. Cardiac fibroblasts were plated in 6-well plates at concentration of $1x10^4$ cells/well in complete growth medium. The following day, cells were incubated with 5 µmol/L CFSE in 1 mL of PBS containing 1% (v/v) FBS for 30 min at 37°C. After the incubation, CFSE-labelled cells were washed twice with PBS and incubated with DMEM/5% FBS or DMEM/5% FBS plus $MgCl_2$ 10 mmol/L. During this period, cells were treated with vehicle or aldosterone (10^{-7} mol/L). Fresh medium with treatments were added every day. After 72 h, cells were harvested in PBS containing 1% FBS and CFSE fluorescence was detected with a FITC channel, using Flow Cytometry (FACS Canto II, BD Biosciences). Data were analyzed using the FlowJo software.

Supplementary References

1 Ryazanova, L. V. *et al.* TRPM7 is essential for Mg(2+) homeostasis in mammals. *Nature communications* **1**, 109, doi:10.1038/ncomms1108 (2010).

2 Rios, F. J. *et al.* Chanzyme TRPM7 protects against cardiovascular inflammation and fibrosis. *Cardiovasc Res*, doi:10.1093/cvr/cvz164 (2019).

3 Kaitsuka, T. *et al.* Inactivation of TRPM7 kinase activity does not impair its channel function in mice. *Scientific reports* **4**, 5718, doi:10.1038/srep05718 (2014).

4 Ryazanova, L. V. *et al.* Elucidating the role of the TRPM7 alpha-kinase: TRPM7 kinase inactivation leads to magnesium deprivation resistance phenotype in mice. *Scientific reports* **4**, 7599, doi:10.1038/srep07599 (2014).

5 Briones, A. M. *et al.* Alterations in structure and mechanics of resistance arteries from ouabain-induced hypertensive rats. *Am J Physiol Heart Circ Physiol* **291**, H193-201, doi:10.1152/ajpheart.00802.2005 (2006).

6 Baumbach, G. L. & Heistad, D. D. Remodeling of cerebral arterioles in chronic hypertension. *Hypertension* **13**, 968-972, doi:10.1161/01.hyp.13.6.968 (1989).

7 Arjona, F. J. *et al.* CNNM2 mutations cause impaired brain development and seizures in patients with hypomagnesemia. *PLoS genetics* **10**, e1004267, doi:10.1371/journal.pgen.1004267 (2014).

8 Livak, K. J. & Schmittgen, T. D. Analysis of relative gene expression data using real-time quantitative PCR and the 2(-Delta Delta C(T)) Method. *Methods* **25**, 402-408, doi:10.1006/meth.2001.1262 (2001).

9 Stritt, S. *et al.* Defects in TRPM7 channel function deregulate thrombopoiesis through altered cellular Mg(2+) homeostasis and cytoskeletal architecture. *Nature communications* **7**, 11097, doi:10.1038/ncomms11097 (2016).

Supplemental table 1. Primer sequences

	Forward 5’-3’	Reverse 5’-3’
TRPM7	TTTGGTGTTCCCAGAAAAGC	ACCAAGTTCCAGGACCACAG
TRPM6	CCTCACGGCTCTACTGAAGG	ACCAGGCTTCCAATGTTGTC
Fibronectin (*FN1*)	CCGGTGGCTGTCAGTCAGA	CCGTTCCCACTGCTGATTTATC
Col1a1	GAGCGGAGAGTACTGGATCG	GACCTCGTGCTCCAGTTAGC
GAPDH	AGGTCGGTGTGAACGGATTTG	TGTAGACCATGTAGTTGAGGTCA

Supplementary Table 2: Values of systolic blood pressure (week 4 of treatment), pD2 and maximal response (Emax%) to acetylcholine (ACh), phenylephrine (Phe), and sodium nitroprusside (SNP) in mesenteric resistant arteries from WT and TRPM7$^{+/\Delta kinase}$.

	WT<br>veh	WT<br>aldo	WT<br>salt	WT<br>aldo/salt	TRPM7+/Δ<br>veh	TRPM7+/Δ<br>aldo	TRPM7+/Δ<br>salt	TRPM7+/Δ<br>aldo/salt
SBP	119.7 ± 3.1<br>N=14	120.5 ± 5.6<br>N=10	120.3 ± 3.6<br>N=7	134.7 ± 3.8*****<br>N=13	115.7 ± 3.2<br>N=17	141.4 ± 5.2†#<br>n=9	135.1 ± 3.5†§<br>n=9	137.9 ± 1.9†<br>n=15
ACh relaxation								
Emax (%)	98.0 ± 4.6	68.4 ± 3.8 *****	97.5 ± 2.8	71.4 ± 2.8 *****	100 ± 2.9	91.6 ± 3.7 †	69.3 ± 3.9 †§	71.7 ± 3.9†
pD2	7.1 ± 0.15	7.2 ± 0.19	7.1 ± 0.10	7.5 ± 0.14	7.6 ± 0.11*****	6.9 ± 0.11†	7.2 ± 0.21†	7.3 ± 0.19†
	N=9	N=10	N=7	N=12	N=10	N=8	N=8	N=12
Phe contraction								
Emax (%)	107 ± 3.0	185 ± 15.7 *****	120 ± 5.7	114 ± 2.9	103 ± 3.3	155 ± 3.5 †	127 ± 7.4	123 ± 2.6 †‡
pD2	6.6 ± 0.07	5.9 ± 0.17	6.2 ± 0.10	6.5 ± 0.06	6.7 ± 0.08	6.0 ± 0.05	5.4 ± 0.10 †§	6.6 ± 0.05
	N=14	N=7	N=7	N=11	N=13	N=8	N=7	N=13
SNP relaxation								
Emax (%)	99.1 ± 2.4	96.7 ± 2.4	102 ± 2.6	78.5 ± 2.7 *****	95.6 ± 2.0	97.2 ± 3.2	109 ± 4.5	84.5 ± 1.6 †
pD2	7.4 ± 0.08	7.7 ± 0.09	7.5 ± 0.09	7.6 ± 0.12	7.9 ± 0.08 *	7.5 ± 0.11†	7.3 ± 0.12 †	7.5 ± 0.06 †
	N=12	N=7	N=7	N=13	N=13	N=8	N=7	N=13

SBP: systolic blood pressure at week 4 of the treatment. Data are expressed as mean ± SEM. ***** $p<0.05$ vs WT veh; † $p<0.05$ vs M7+/Δ veh; ‡$p<0.05$ M7+/Δ aldo-salt vs WT aldo-salt; # $p<0.05$ M7+/Δ aldo vs WT aldo; §$p<0.05$ M7+/Δ salt vs WT salt.

Supplementary Figure 1

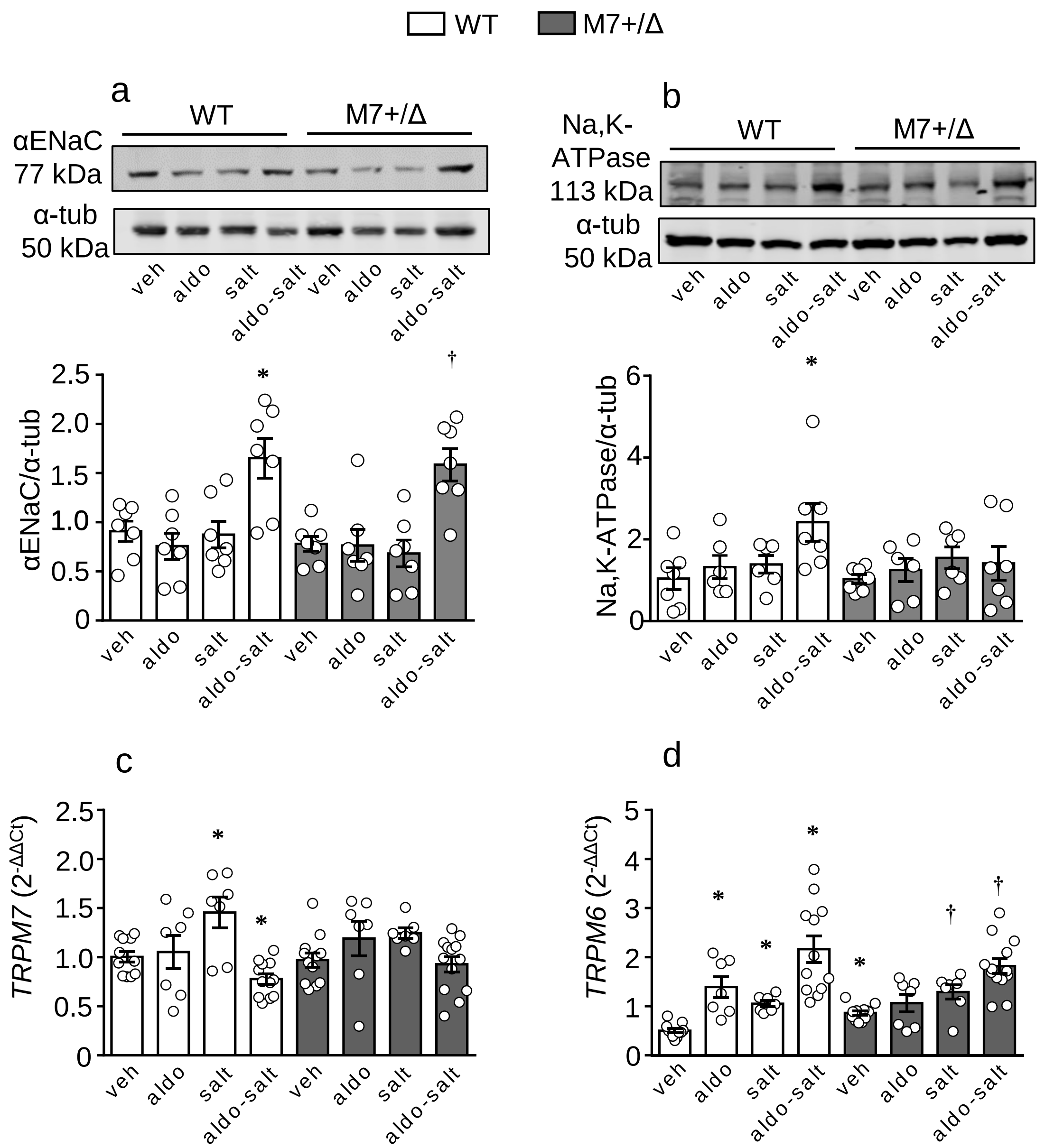

Supplementary Figure 1. Expression of αENaC, Na,K-ATPase, TRPM7 and TRPM6. Total protein and RNA were isolated from kidney tissues, WT (white bars) $TRPM7^{+/\Delta kinase}$ (M7+/Δ, grey bars). Protein expression for (a) αENaC and (b) Na,K-ATPase was assessed by immunoblotting and normalized by α-tubulin. Gene expression for (c) *TRPM7* and (d) *TRPM6* was determined by real-time PCR and normalized by *GAPDH*. N numbers: WT (veh=11, aldo=7, salt=7, aldo-salt=12), M7+/Δ (veh=12, aldo=7, salt=7, aldo-salt=13). Data are expressed as mean ± SEM of $2^{-\Delta\Delta Ct}$ values. One-way ANOVA followed by Dunnett's multiple comparisons test were used for statistical analysis. *P<0.05 vs WT veh; † P<0.05 vs M7+/Δ veh.

Supplementary Figure 2

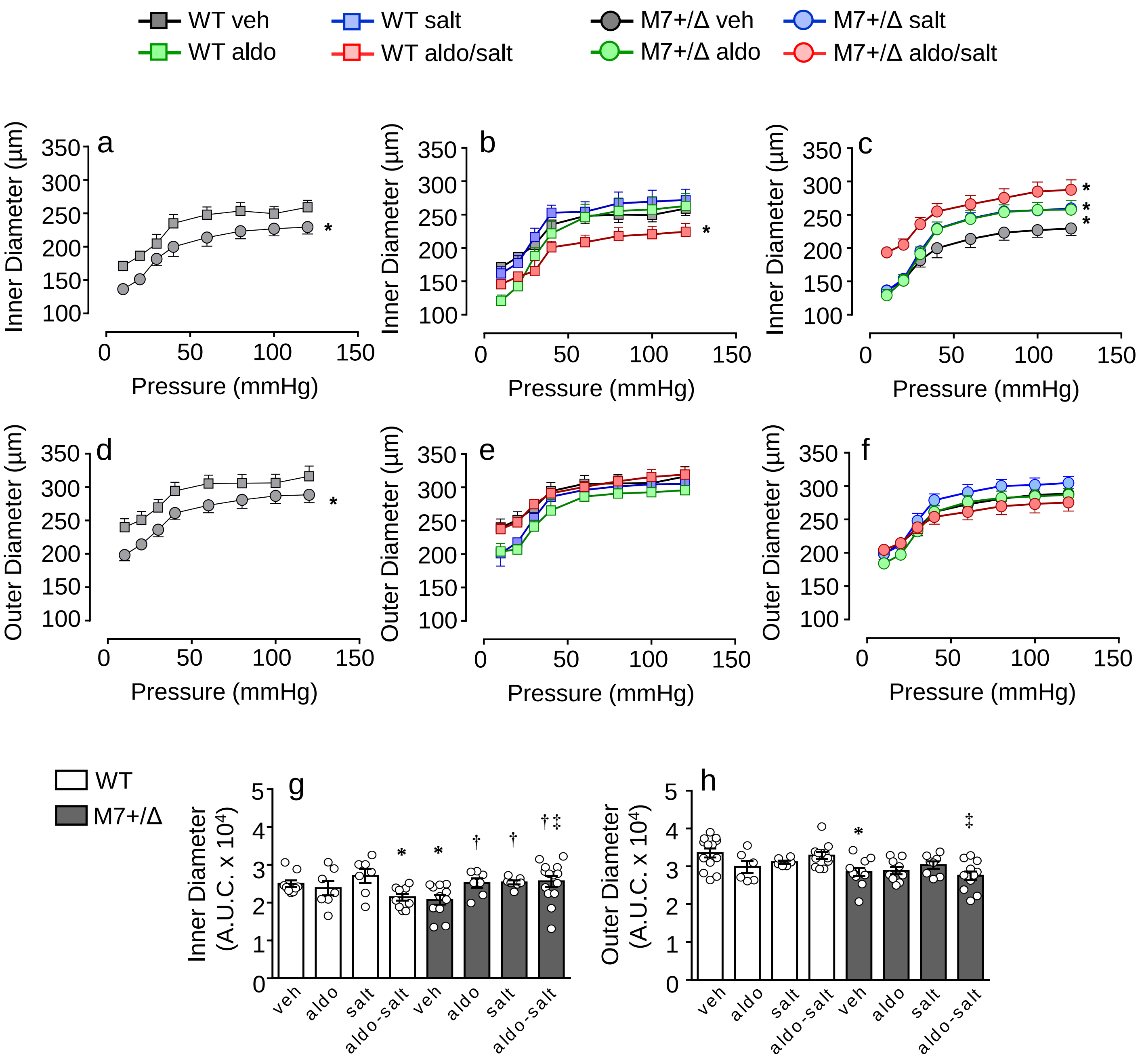

Supplementary Figure 2. Vascular structure of mesenteric vessels from WT and TRPM7$^{+/\Delta kinase}$ mice. Animals WT (squares) and TRPM7$^{+/\Delta kinase}$ (M7+/Δ) (circles) were treated with aldosterone (aldo, green), high salt (salt, blue) or aldosterone-salt (aldo-salt, red). Vascular structure was assessed in pressurized mesenteric arteries at increasing intraluminal pressure (10–120 mmHg) in calcium-free conditions. (a-c) inner diameter and (d-f) outer diameter; (g, h) Area under the curve (A.U.C.) of inner and outer diameter respectively. N numbers: WT (veh=11; aldo=7; salt=7; aldo-salt=11), M7+/Δ (veh=11; aldo=7; salt=7; aldo-salt=14). Data are present as mean ± SEM. One-way ANOVA followed by Dunnett's multiple comparisons test were used for statistical analysis. WT (white bars) and M7+/Δ (grey bars). *P<0.05 vs WT veh; †p<0.05 vs M7+/Δ veh. ‡M7+/Δ aldo-salt vs WT aldo-salt.

Supplementary Figure 3

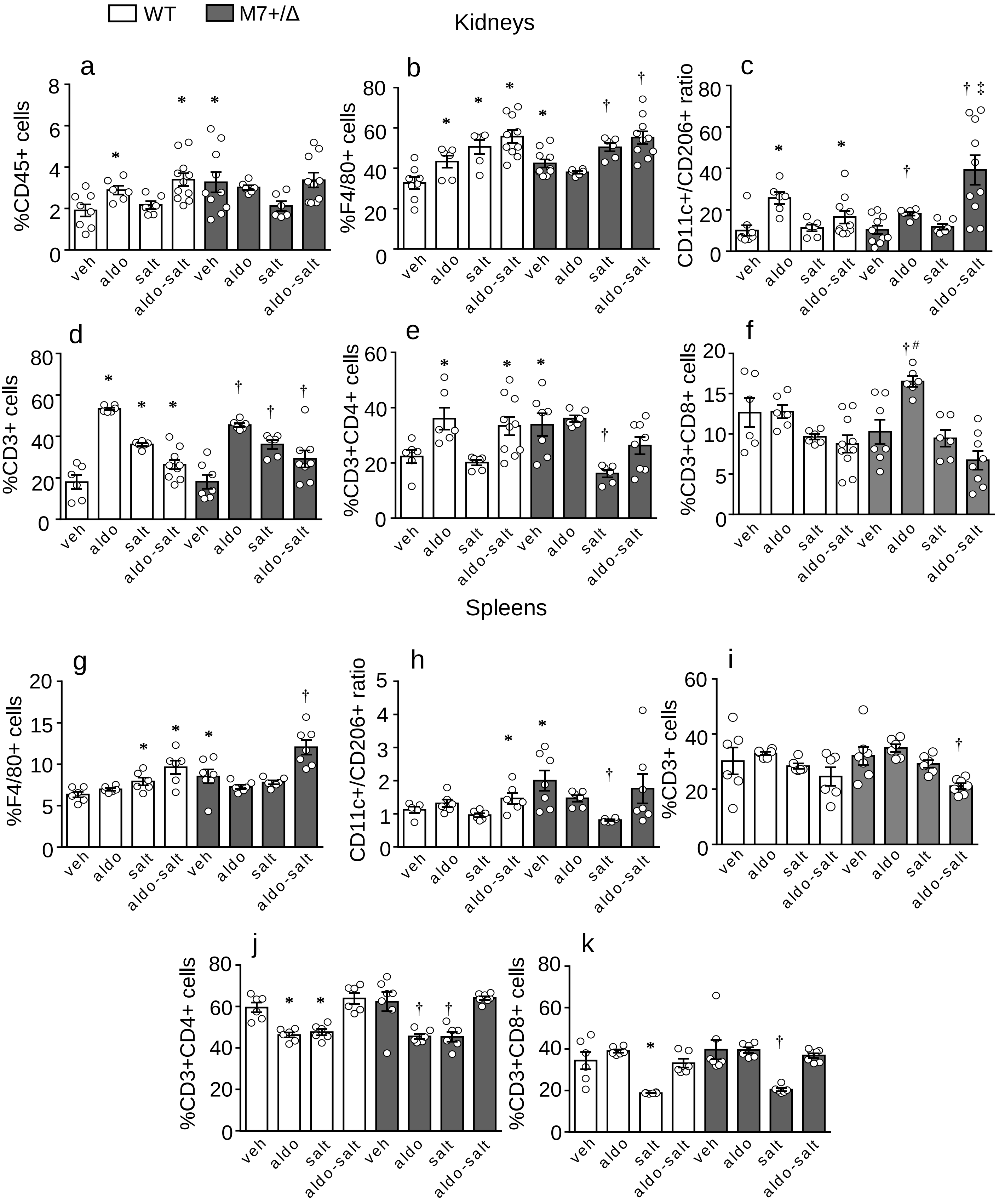

Supplementary Figure 3. Immune cell profile in kidneys and spleens. (a-f) Cells were isolated from kidneys and stained for flow cytometry analysis: (a) CD45+ population (total hematopoietic cells); (b) CD45+F4/80+ cells (macrophages); (c) CD11+/CD206+ ratio; (d) CD45+CD3+ cells (T lymphocytes); (e) CD45+CD3+CD4+ cells (CD4 T lymphocytes) (f) CD45+CD3+CD8+ cells (CD8 T lymphocytes). WT white bars (V=6-8; A=6; S=6; A-S=10); M7+/Δ grey bars (V=7-10, A=6, S=6, A-S=8-10)]. (g-k) Cells were isolated from spleens: (g) CD45+F4/80+ cells (macrophages); (h) CD11+/CD206+ ratio; (i) CD45+F4/80+CD3+ cells (T lymphocytes); (j) CD45+CD3+CD4+ cells (CD4 T lymphocytes) (k) CD45+CD3+CD8+ cells (CD8 T lymphocytes). WT (veh=6, aldo=6, salt=6, aldo-salt=6); M7+/Δ (veh=7, aldo=6, salt=6, aldo-salt=7). Results are shown as mean ± SEM. One-way ANOVA followed by Dunnett's multiple comparisons test were used for statistical analysis. *$P<0.05$ vs WT veh; †$p<0.05$ vs TRPM7$^{+/\Delta kinase}$ (M7+/Δ) veh.

Supplementary Figure 4

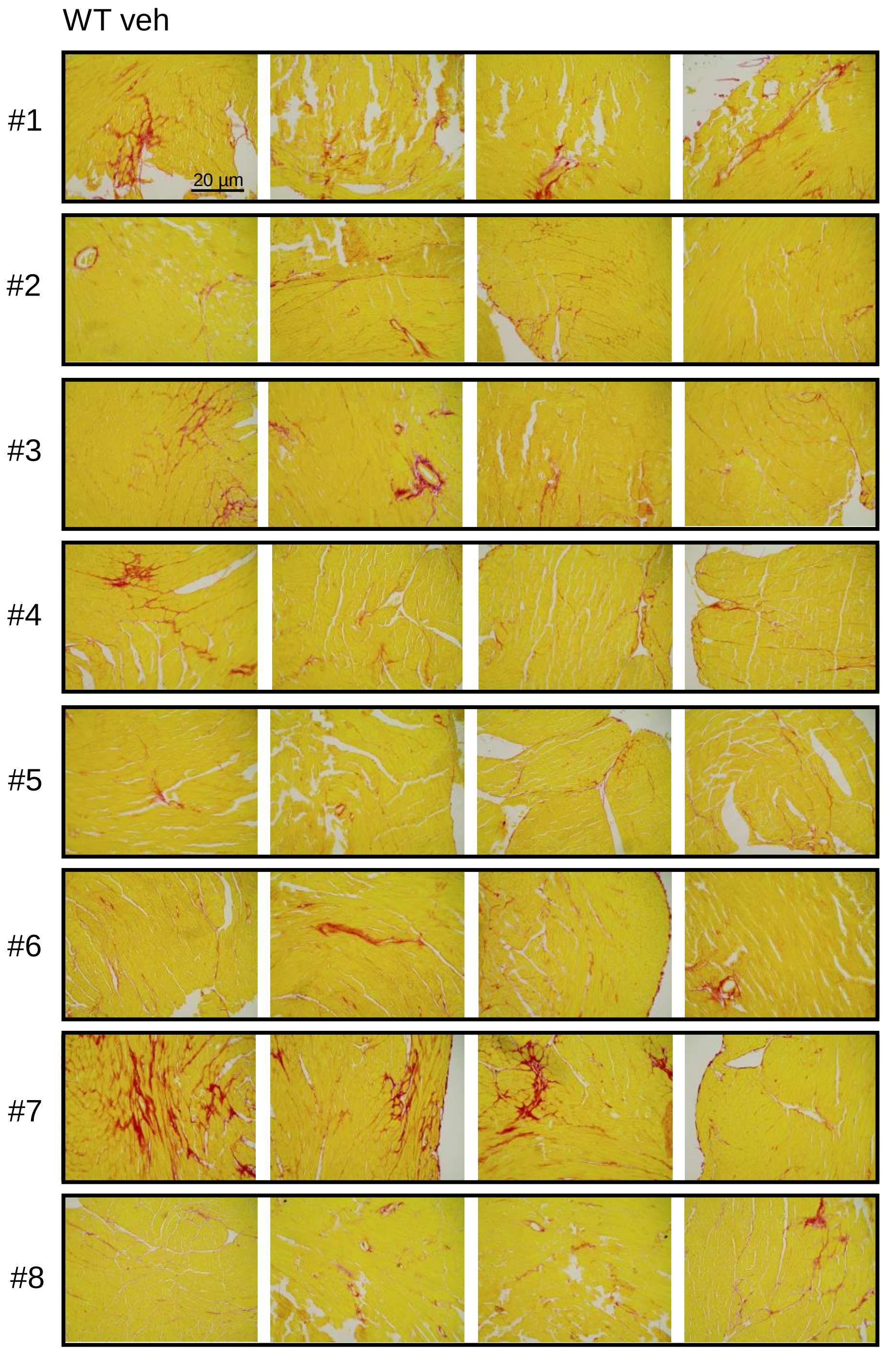

Supplementary Figure 4. Collagen deposition in hearts from WT vehicle treated mice. Cardiac tissues from WT mice were stained with picro-sirius red. Collagen content was assessed in bright field microscopy (scale bar 20 µm). Each panel shows 4 representative images from each animal (n=8).

Supplementary Figure 5

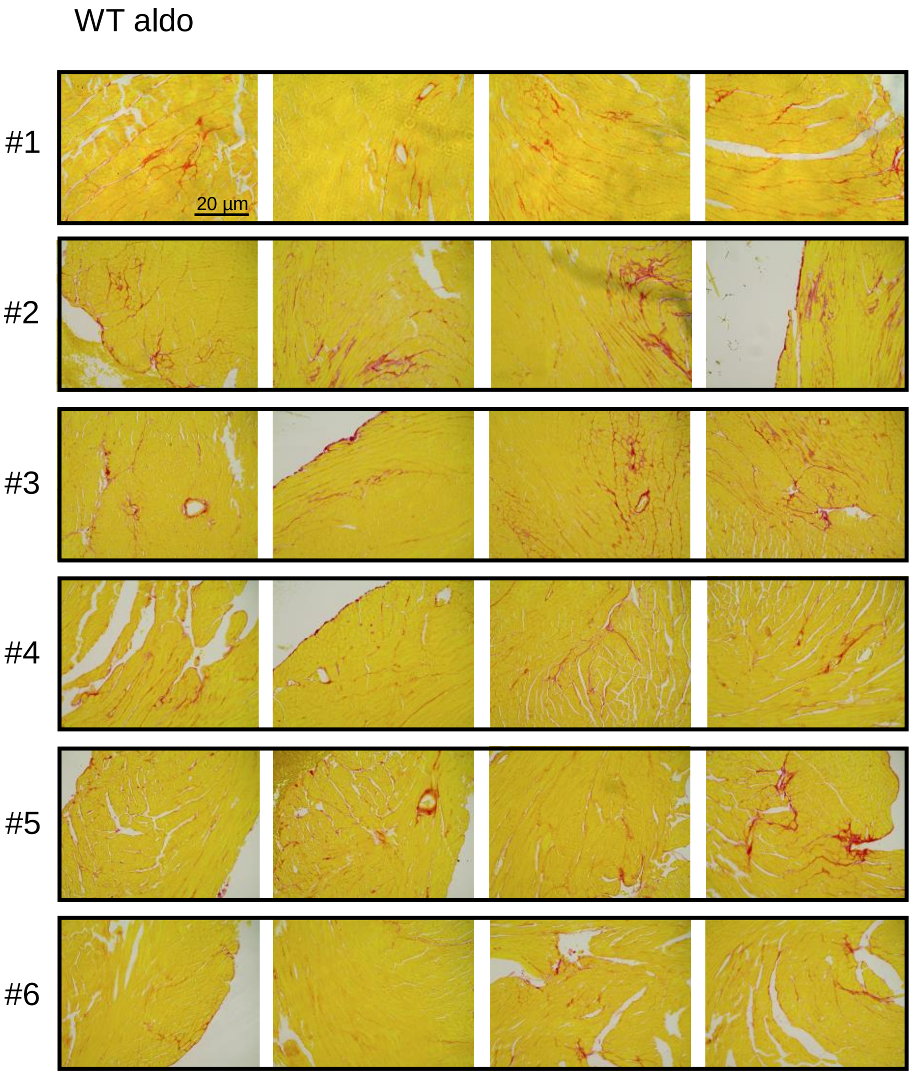

Supplementary Figure 5. Collagen deposition in hearts from WT mice treated with aldosterone. Cardiac tissues were stained with picro-sirius red. Collagen content was assessed in bright field microscopy (scale bar 20 µm). Each panel shows 4 representative images from each animal (n=6).

Supplementary Figure 6

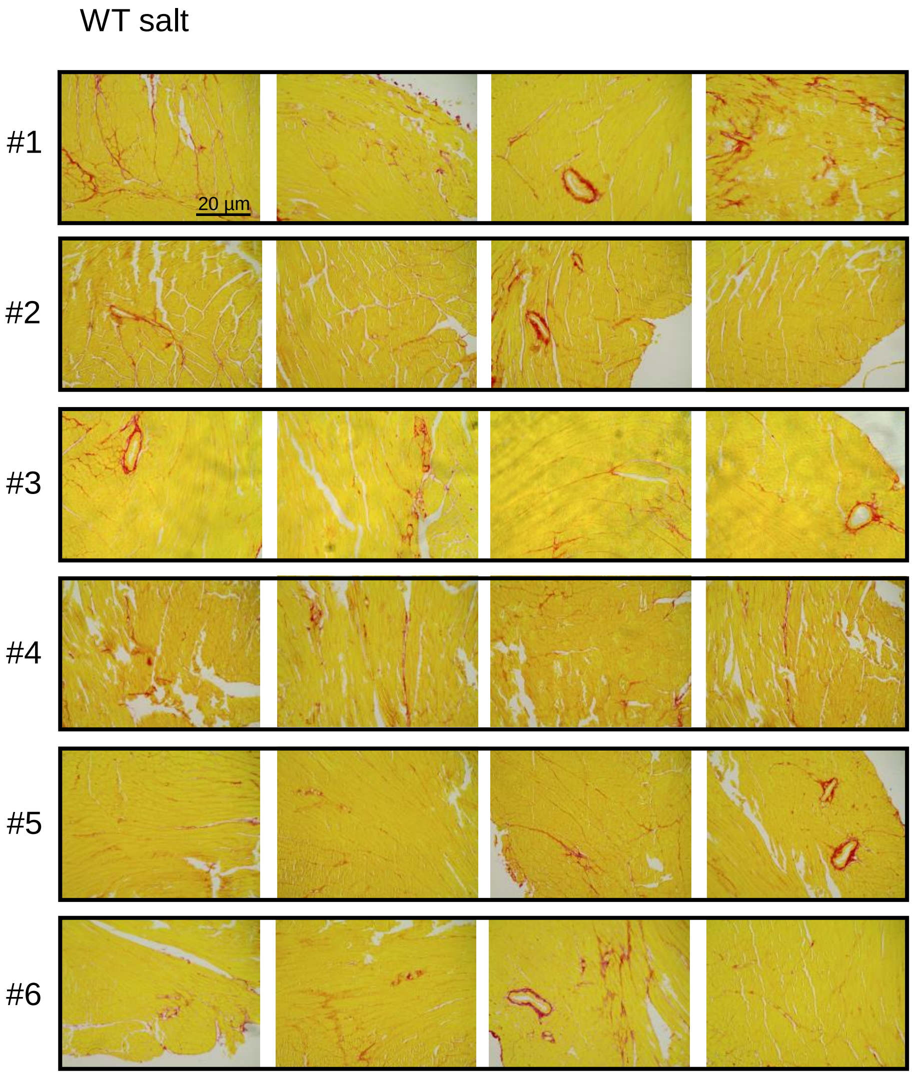

Supplementary Figure 6. Collagen deposition in hearts from WT mice treated with salt. Cardiac tissues were stained with picro-sirius red. Collagen content was assessed in bright field microscopy (scale bar 20 µm). Each panel shows 4 representative images from each animal (n=6).

Supplementary Figure 7

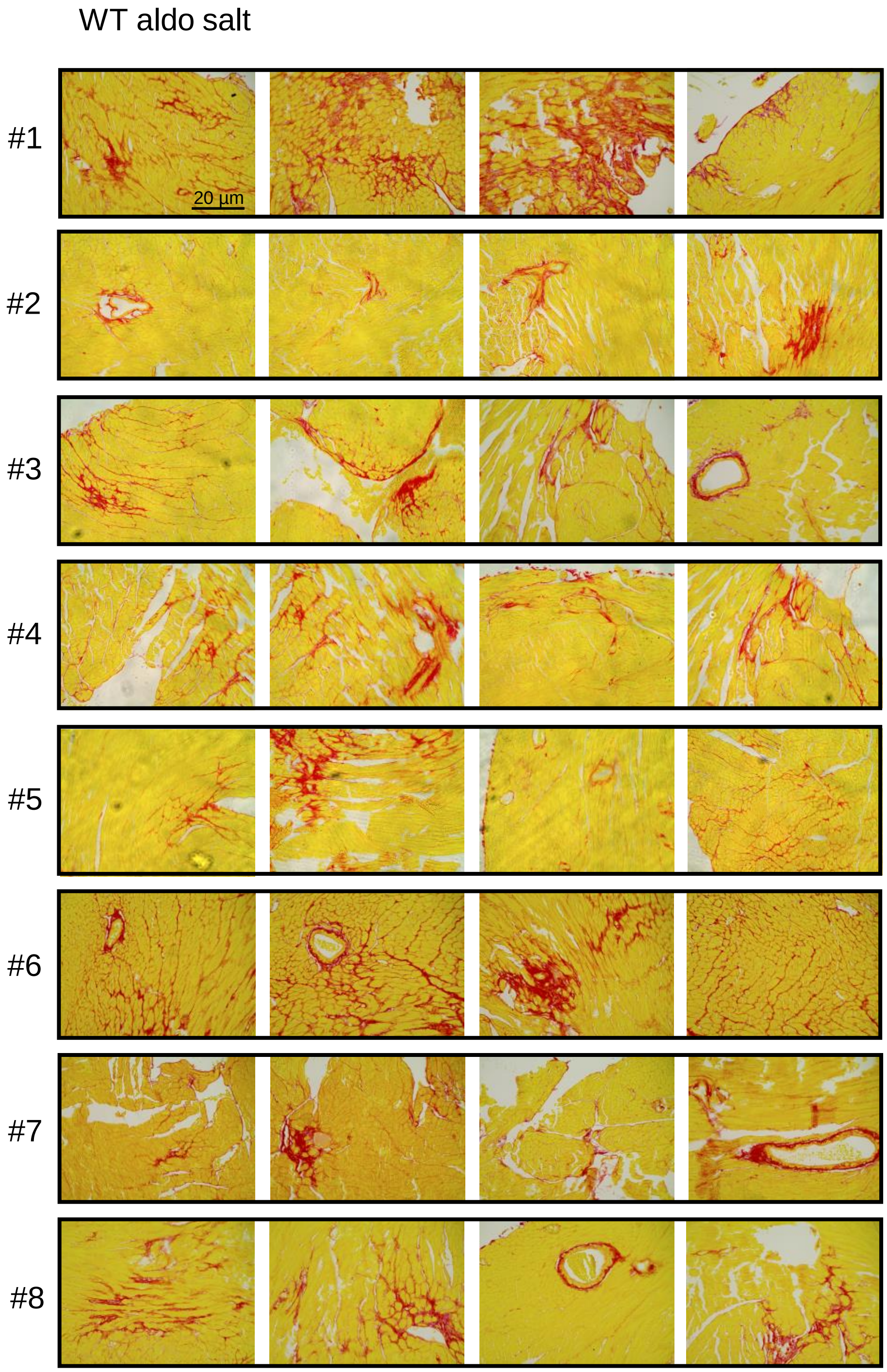

Supplementary Figure 7. Collagen deposition in hearts from WT mice treated with aldosterone and salt. Cardiac tissues were stained with picro-sirius red. Collagen content was assessed in bright field microscopy (scale bar 20 µm). Each panel shows 4 representative images from each animal (n=8).

Supplementary Figure 8

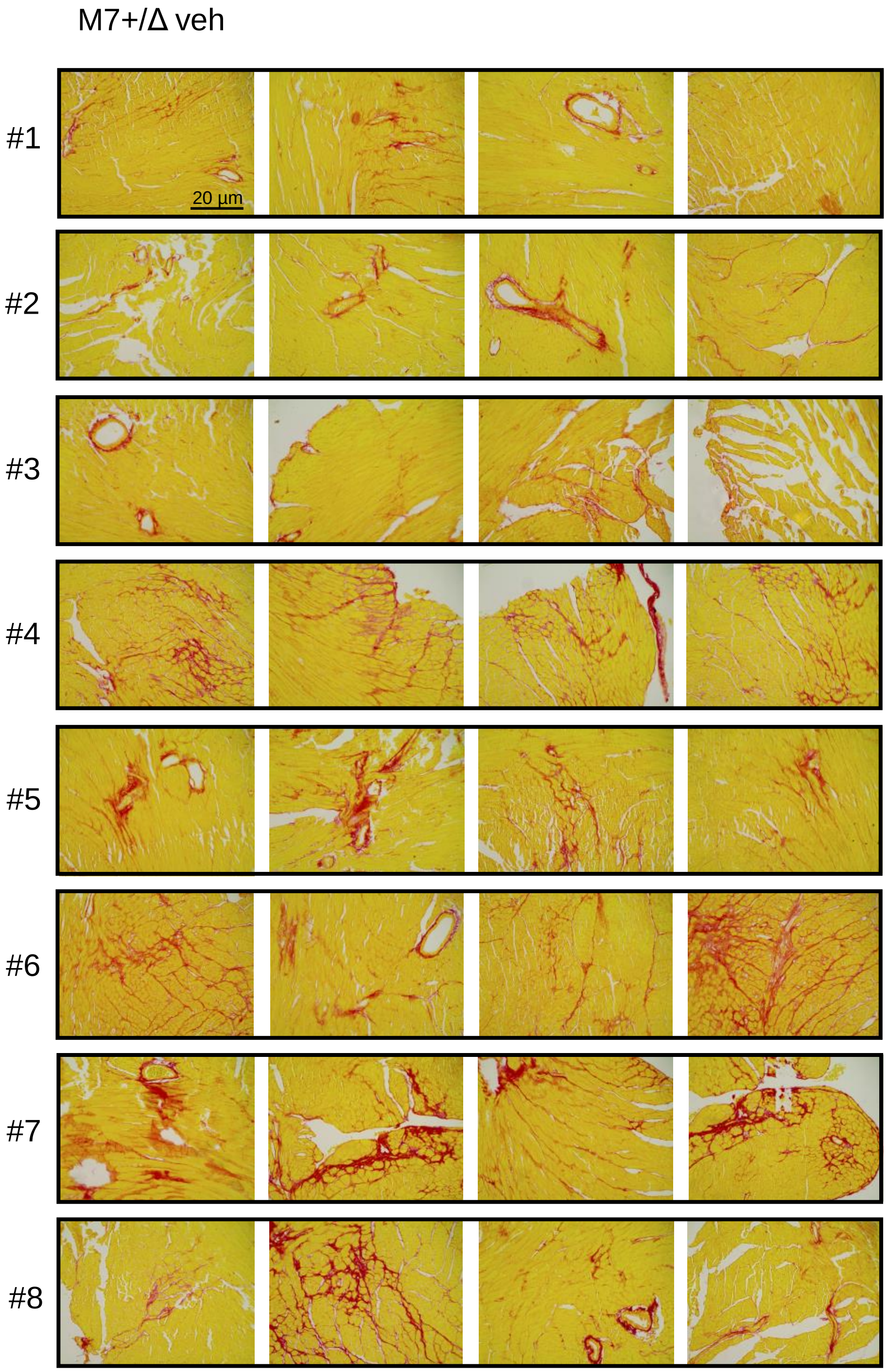

Supplementary Figure 8. Collagen deposition in hearts from TRPM7$^{+/\Delta kinase}$ vehicle treated mice. Cardiac tissues were stained with picro-sirius red. Collagen content was assessed in bright field microscopy (scale bar 20 µm). Each panel shows 4 representative images from each animal (n=8).

Supplementary Figure 9

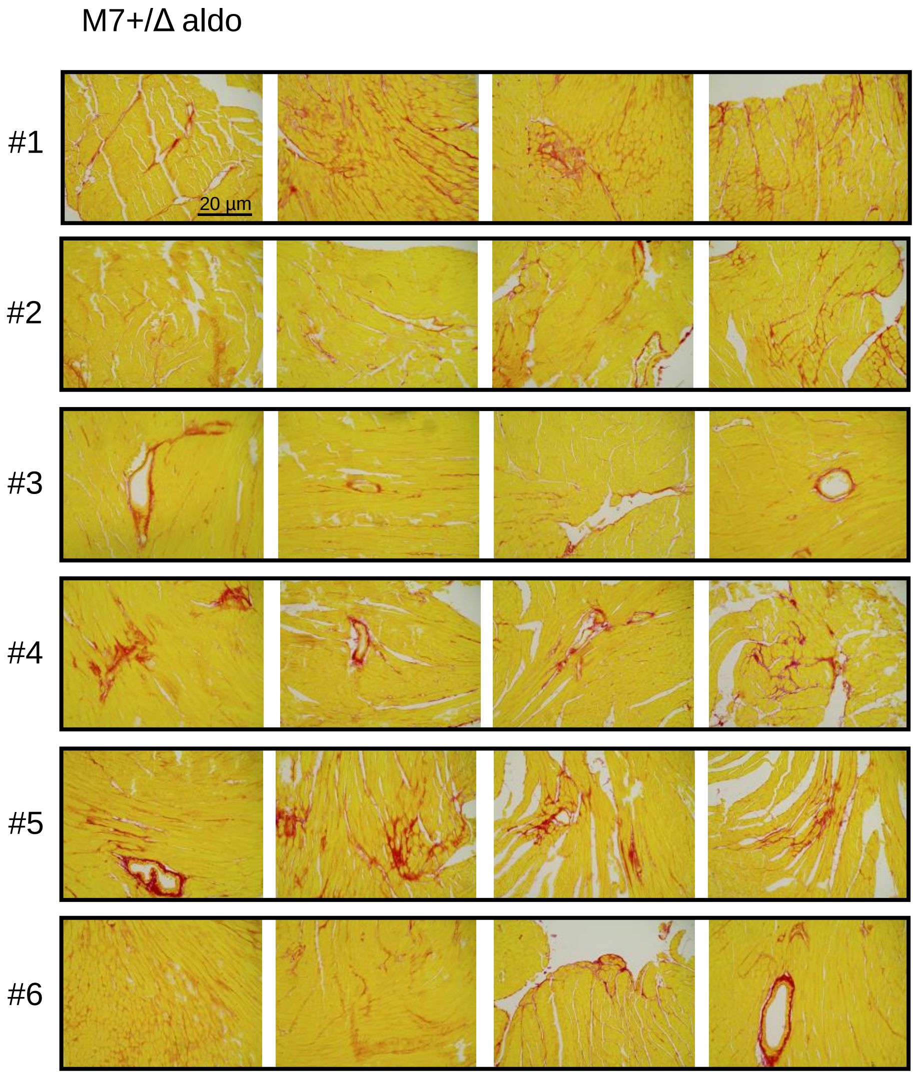

Supplementary Figure 9. Collagen deposition in hearts from TRPM7$^{+/\Delta kinase}$ mice treated with aldosterone. Cardiac tissues were stained with picro-sirius red. Collagen content was assessed in bright field microscopy (scale bar 20 µm). Each panel shows 4 representative images from each animal (n=6).

Supplementary Figure 10

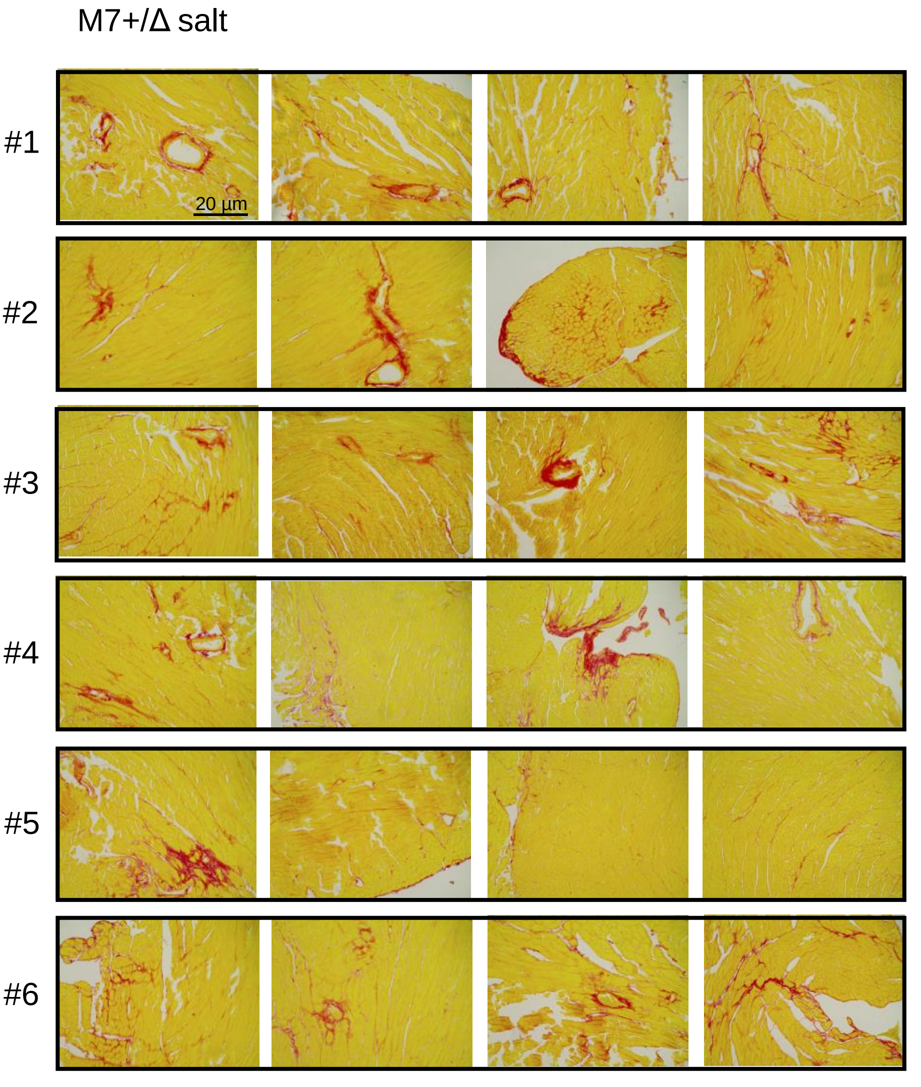

Supplementary Figure 10. Collagen deposition in hearts from TRPM7$^{+/\Delta kinase}$ mice treated with salt. Cardiac tissues were stained with picro-sirius red. Collagen content was assessed in bright field microscopy (scale bar 20 µm). Each panel shows 4 representative images from each animal (n=6).

Supplementary Figure 11

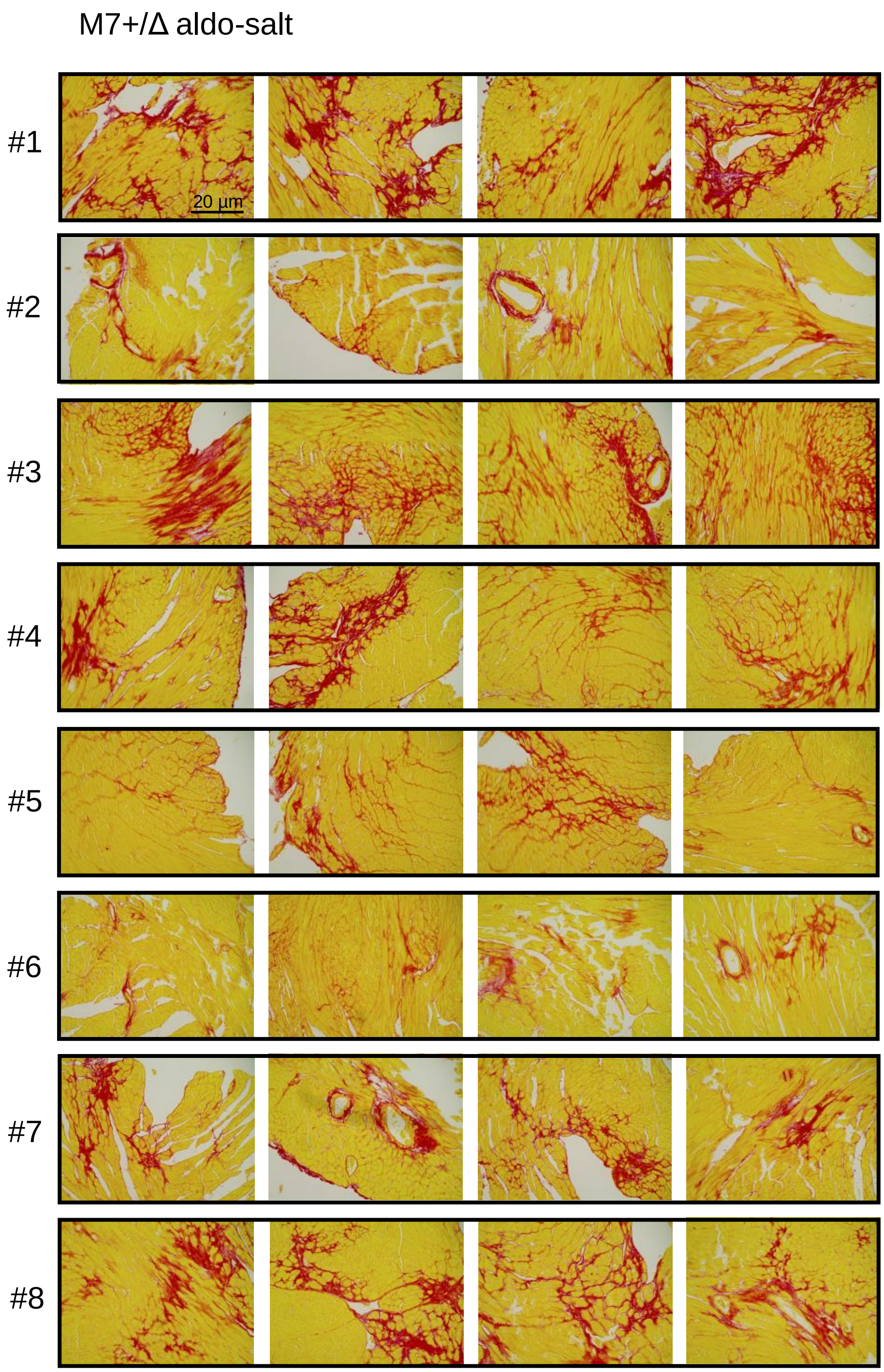

Supplementary Figure 11. Collagen deposition in hearts from TRPM7$^{+/\Delta kinase}$ mice treated with aldosterone and salt. Cardiac tissues were stained with picro-sirius red. Collagen content was assessed in bright field microscopy (scale bar 20 µm). Each panel shows 4 representative images from each animal (n=8).

Supplementary Figure 12

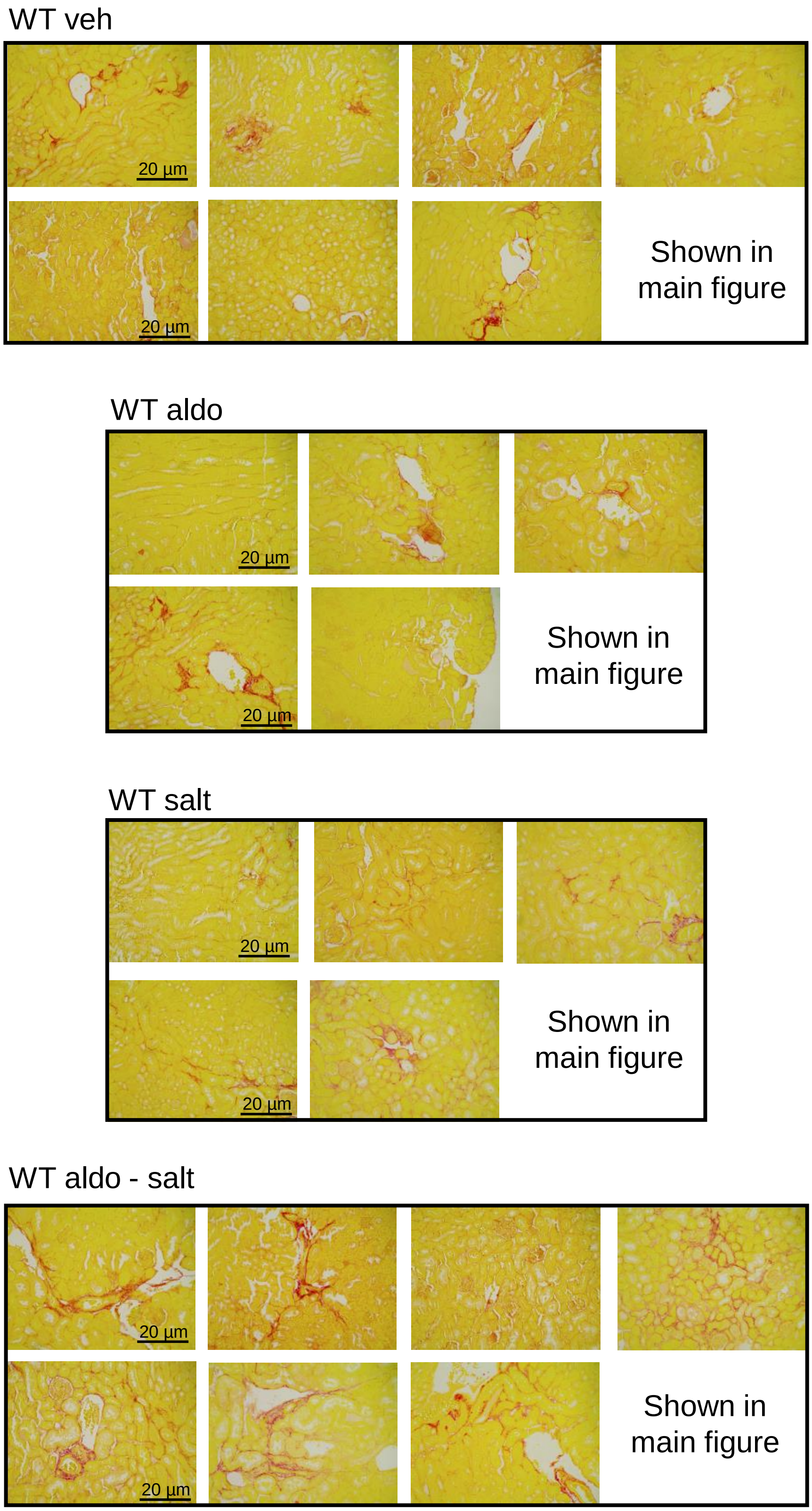

Supplementary Figure 12. Collagen deposition in kidneys from WT mice treated with aldosterone and/or salt. Tissues from WT mice were stained with picro-sirius red. Collagen content was assessed in bright field microscopy (scale bar 20 µm). Each panel shows representative figures from each animal. N numbers: WT (veh=8, aldo=6, salt=6, aldo-salt=8).

Supplementary Figure 13

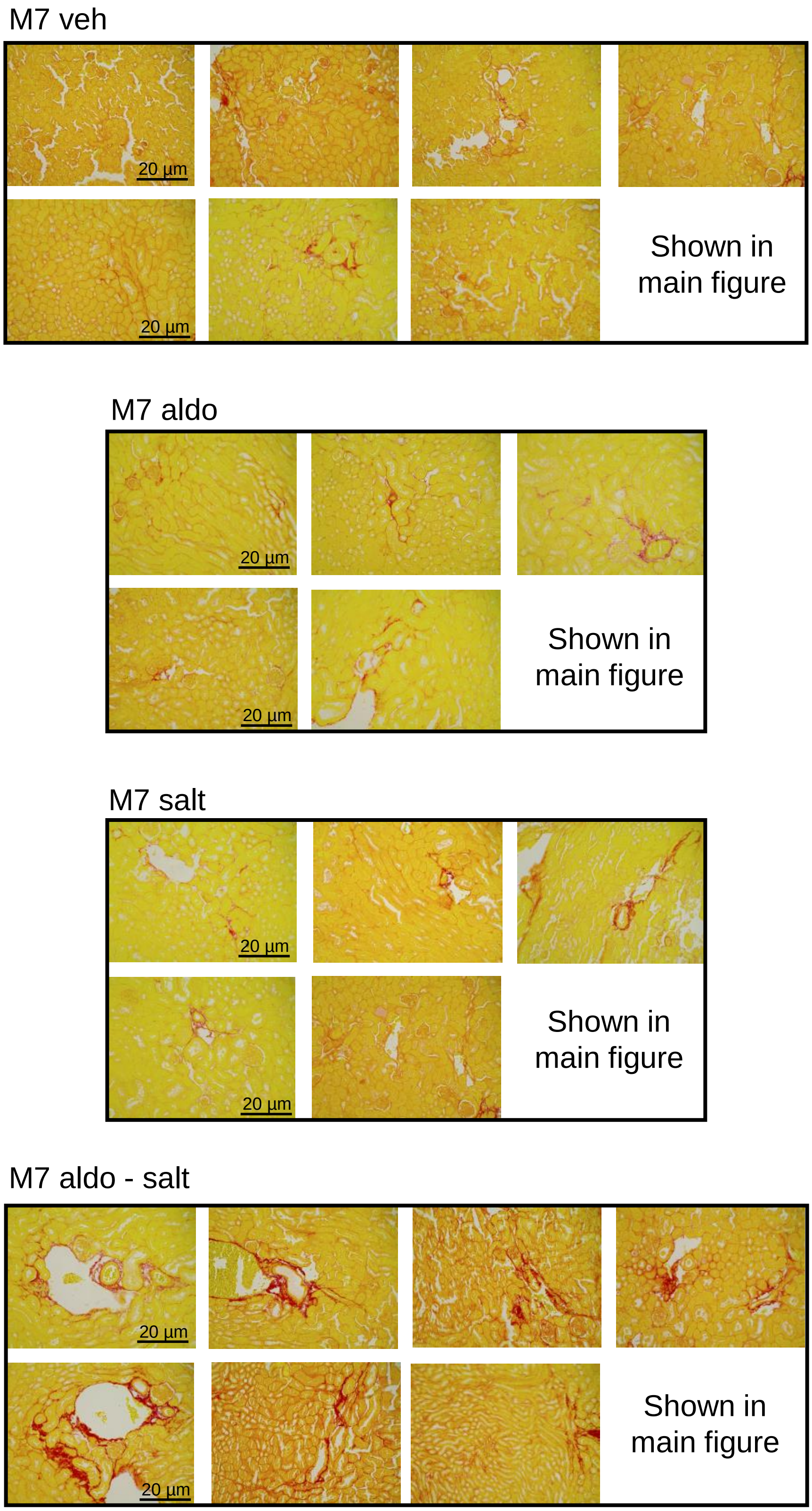

Supplementary Figure 13. Collagen deposition in kidneys from TRPM7$^{+/\Delta kinase}$ mice treated with aldosterone and/or salt. Tissues from TRPM7$^{+/\Delta kinase}$ mice were stained with picro-sirius red. Collagen content was assessed in bright field microscopy (scale bar 20 µm). Each panel shows representative figures from each animal. N numbers: WT (veh=8, aldo=6, salt=6, aldo-salt=8).

Supplementary Figure 14

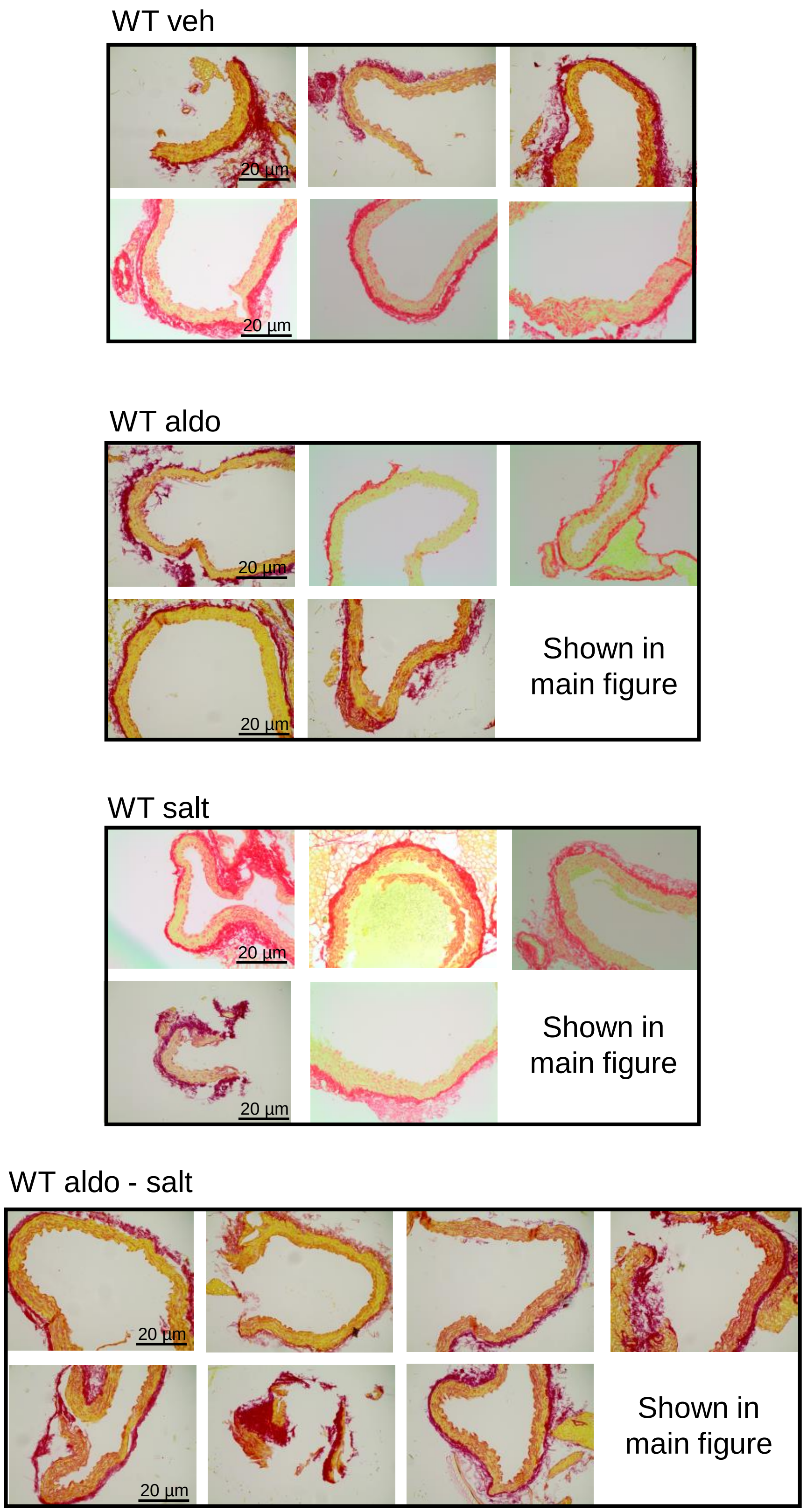

Supplementary Figure 14. Collagen deposition in Aortas from WT mice treated with aldosterone and/or salt. Tissues from WT mice were stained with picro-sirius red. Collagen content was assessed in bright field microscopy (scale bar 20 µm). Each panel shows representative figures from each animal. N numbers: WT (veh=7, aldo=6, salt=6, aldo-salt=7).

Supplementary Figure 15

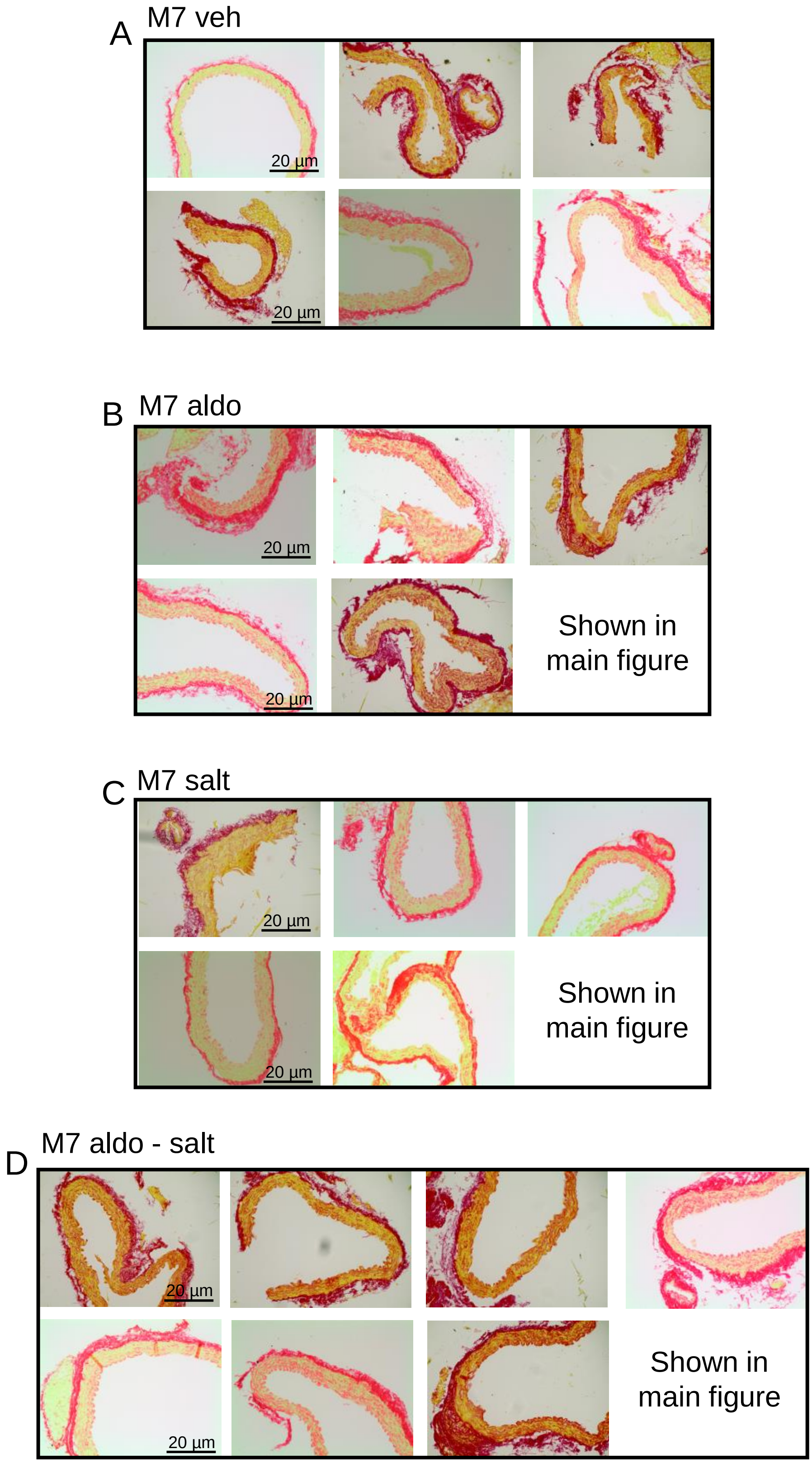

Supplementary Figure 15. Collagen deposition in kidneys from TRPM7$^{+/\Delta kinase}$ mice treated with aldosterone and/or salt. Tissues from WT mice were stained with picro-sirius red. Collagen content was assessed in bright field microscopy (scale bar 20 µm). Each panel shows representative figures from each animal. N numbers: WT (veh=8, aldo=6, salt=6, aldo-salt=8).

Supplementary Figure 16

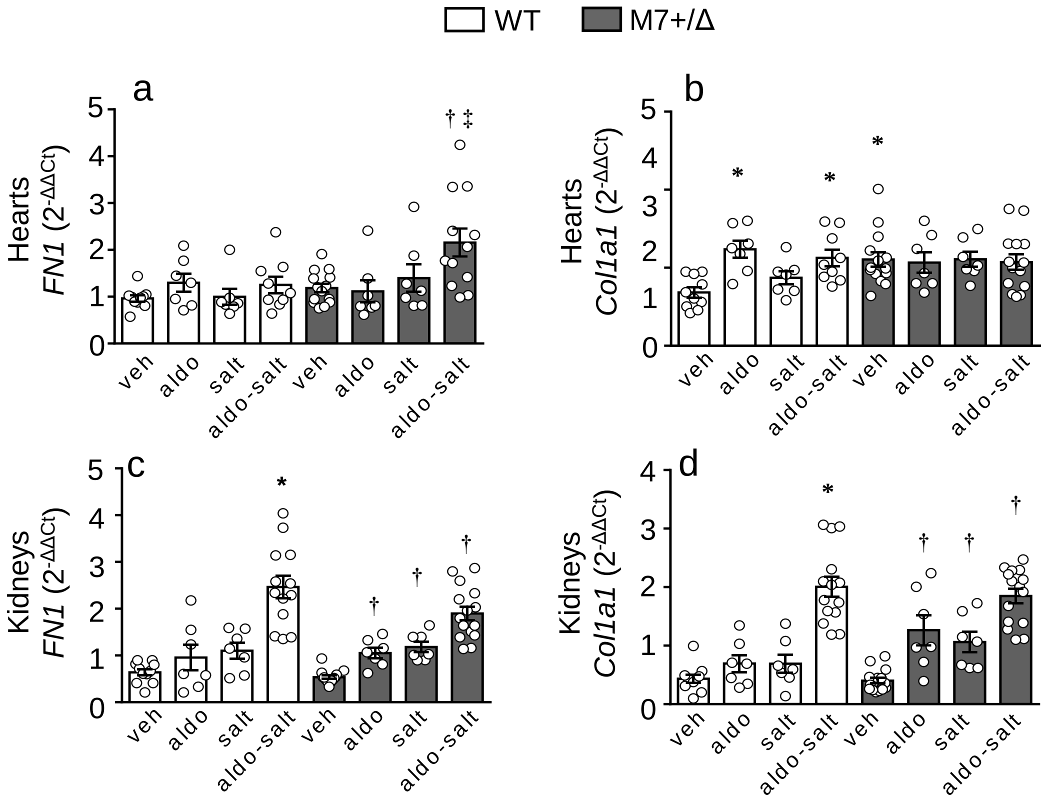

Supplementary Figure 16. Fibrotic markers in hearts and kidneys from WT and TRPM7$^{+/\Delta kinase}$ mice. Total RNA was extracted from tissues, gene expression was determined by real-time PCR and normalized by *GAPDH*. (a) Fibronectin (*FN1*) and (b) collagen-1 (*Col1a1*) in hearts [WT (veh=10, aldo=7, salt=7, aldo/salt=9) M7+/Δ (veh=14, aldo=7, salt=7, aldo/salt=14)]. (c) *FN1* and (d) *Col1a1* in kidneys [WT (veh=11, aldo=7, salt=7, aldo/salt=14) M7+/Δ (veh=15, aldo=7, salt=7, aldo/salt=15)]. Data are expressed in mean ± SEM of $2^{-\Delta\Delta Ct}$ values. One-way ANOVA followed by Dunnett's multiple comparisons test were used for statistical analysis. *P<0.05 vs WT veh; †p<0.05 vs TRPM7$^{+/\Delta kinase}$ (M7+/Δ) veh; ‡M7+/Δ aldo-salt vs WT aldo-salt

Supplementary Figure 17

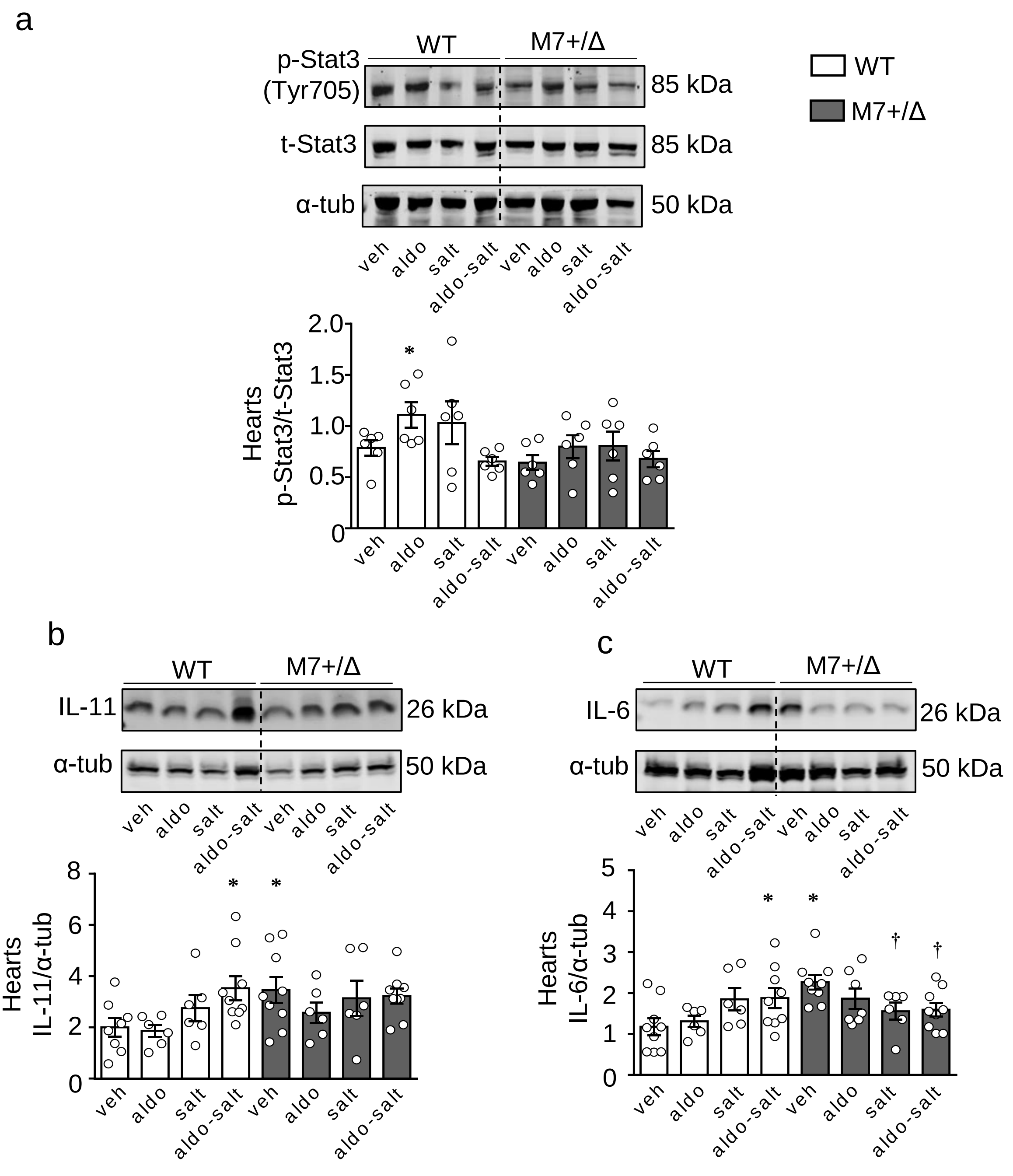

Supplementary Figure 17. Cardiac expression of phospho-Stat3, IL-11 and IL-6. Heart tissues (n=6-9/group) were analyzed by immunoblotting for protein expression of (a) phospho-Stat3(Tyr705), (b) IL-11 and (c) IL-6. Total protein expression was normalized total-Stat3 or α-tubulin as indicated. Data are expressed as mean± SEM and representative figures. WT, white bars and M7+/Δ, grey bars. One-way ANOVA followed by Dunnett's multiple comparisons test were used for statistical analysis. *P<0.05 vs WT veh; †p<0.05 vs M7+/Δ veh.

Supplementary Figure 18

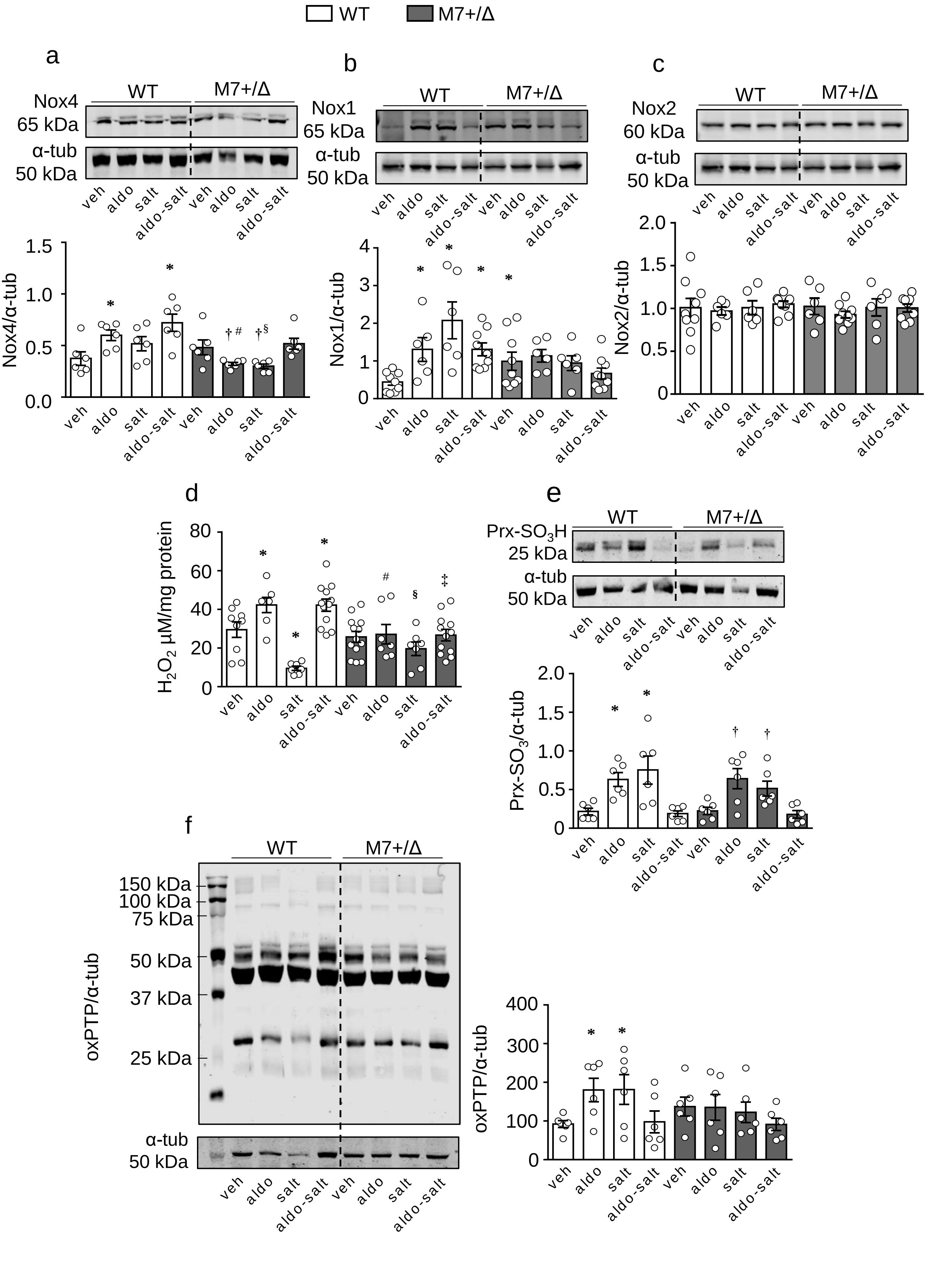

Supplementary Figure 18. Noxs expression, H_2O_2 production and protein oxidation in hearts tissues. Cardiac tissues from WT (white bars) and TRPM7$^{+/\Delta kinase}$ (M7+/Δ, grey bars) were investigated for protein expression of (a) Nox4, (b) Nox1 and (c) Nox2, (d) H_2O_2 production, (e) expression of oxidized form of peroxiredoxin (Prx-SO_3H) and (F) expression of oxidized protein tyrosine phosphatase (oxPTP). Data were normalized by α-tubulin (n=6/group). H_2O_2 production was accessed by Amplex Red assay and normalized by protein concentration. WT (veh=9, aldo=7, salt=7, aldo-salt=12) M7+/Δ (veh=13, aldo=7, salt=7, aldo-salt=12). Data are presented as representative figures and mean ± SEM. One-way ANOVA followed by Dunnett's multiple comparisons test were used for statistical analysis. *P<0.05 compared with WT veh. †p<0.05 compared with M7+/Δ veh. ‡M7+/Δ aldo-salt *vs* WT aldo/salt; $^{\#}$P<0.05 M7+/Δ aldo *vs* WT aldo; § M7+/Δ salt *vs* WT salt.

Supplementary Figure 19

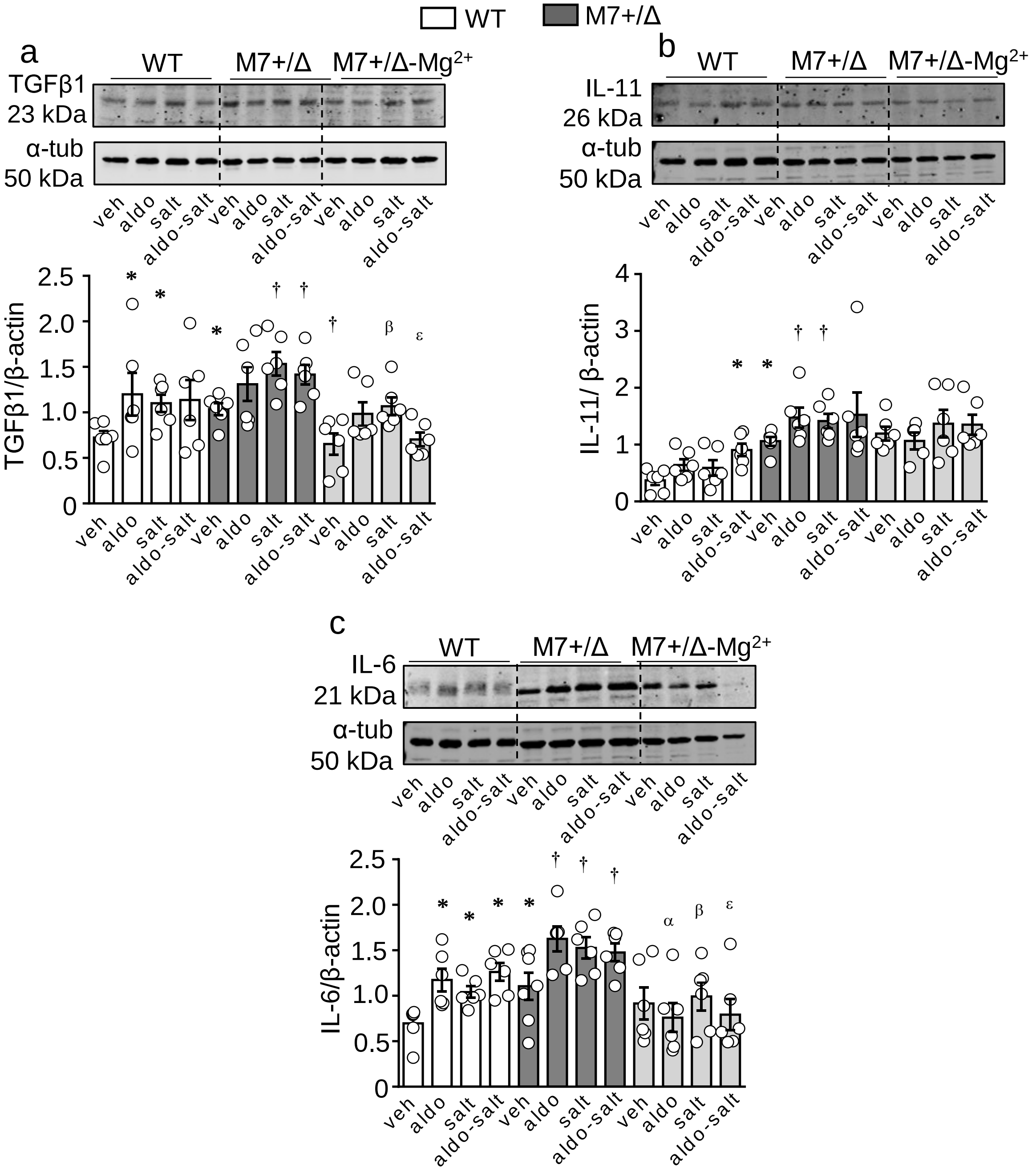

Supplementary Figure 19. Expression of TGFβ1, IL-11 and IL-6 in cardiac fibroblasts from WT and TRPM7$^{+/\Delta kinase}$ mice. Cardiac fibroblasts were isolated from WT and TRPM7$^{+/\Delta kinase}$ (M7+/Δ) animals. Part of the cells from TRPM7$^{+/\Delta kinase}$ (M7+/Δ) animals were constantly treated with 10 mM of $MgCl_2$. 24 h before the experiments, medium was changed to 0.5% FBS and part of the cells were treated with NaCl 40 mM or osmotic control Choline Chloride (40 mM). Cells were stimulated with aldosterone (10^{-7} mmol/L) for 24 h. Protein expression of (a) TGFβ1, (b) IL-11 and (c) IL-6 was assessed by immunoblotting and normalized to α-tubulin. Data are expressed as mean ± SEM and representative figures (n=7/group). One-way ANOVA followed by Dunnett's multiple comparisons test were used for statistical analysis. *P<0.05 compared with WT veh. *P<0.05 vs WT veh. †p<0.05 vs with M7+/Δ veh. $^{\alpha}$ p<0.05 M7+/Δ-Mg^{2+} aldo vs M7+/Δ aldo. $^{\beta}$p<0.05 M7+/Δ-Mg^{2+} salt vs M7+/Δ salt. $^{\varepsilon}$ p<0.05 M7+/Δ-Mg^{2+} aldo-salt vs M7+/Δ aldo-salt.

Supplementary Figure 20

Figure 1a – pTRPM7

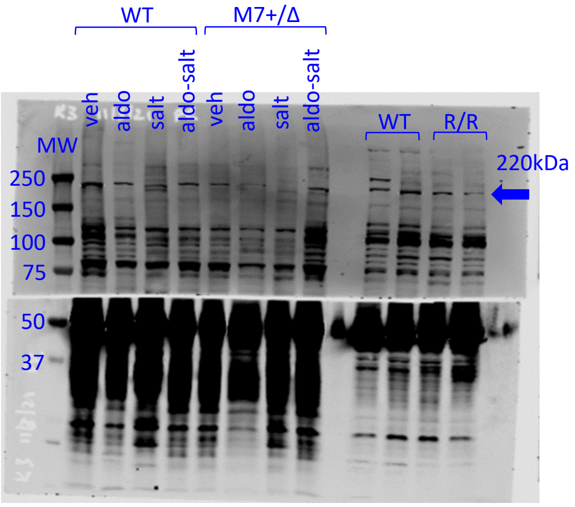

Figure 1a- α-tubulin

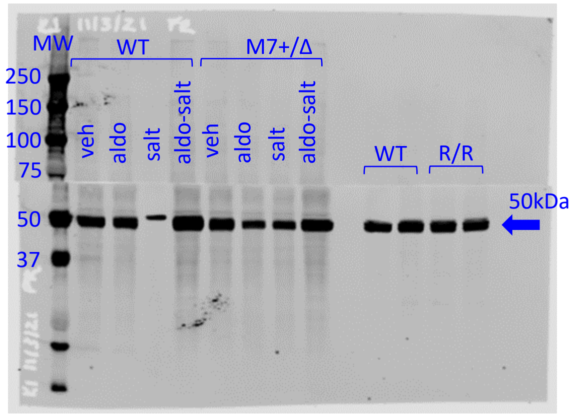

Figure 1b - TRPM7

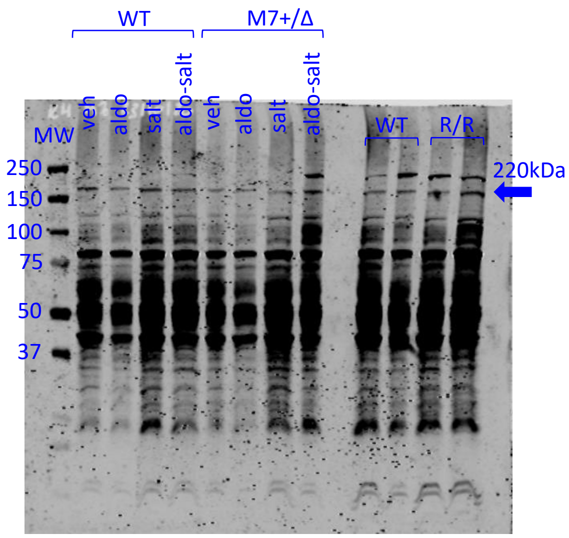

Figure 1b - α-tubulin

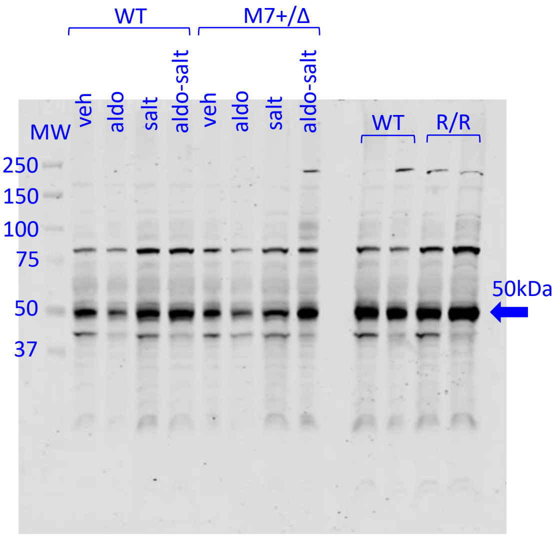

Supplementary Figure 21

Figure 5a – Hearts PPM1A

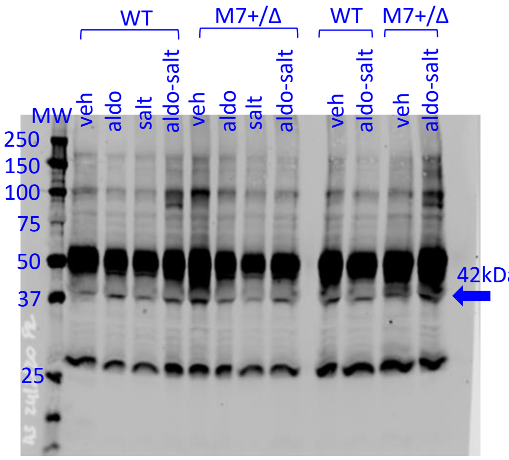

Figure 5a – Hearts - α-tubulin

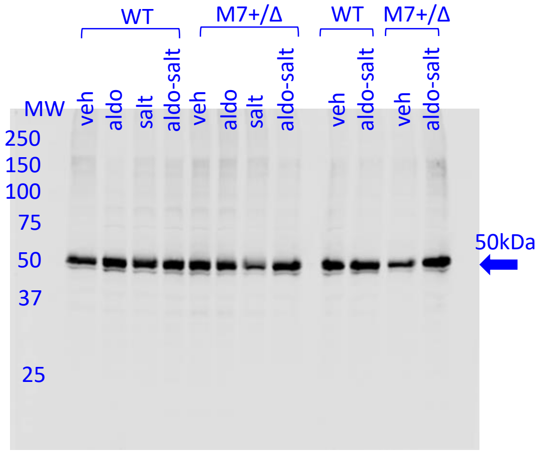

Figure 5b – Kidneys – PPM1A

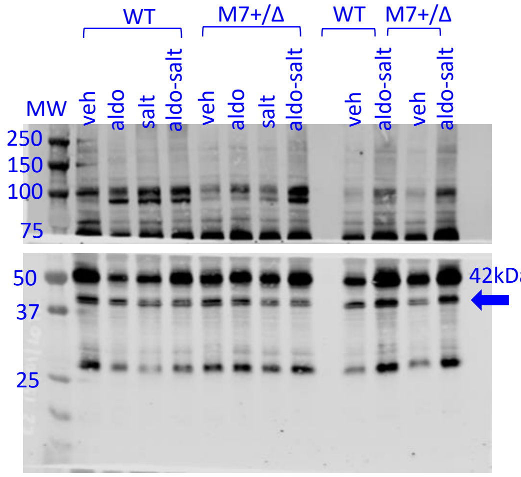

Figure 5b – Kidneys – α-tubulin

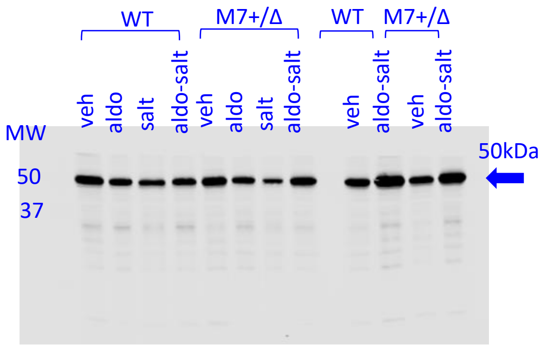

Figure 5c – Aortas – PPM1A

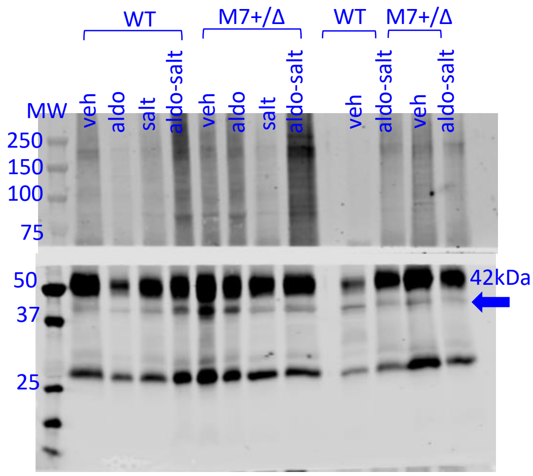

Figure 5c – Kidneys – α-tubulin

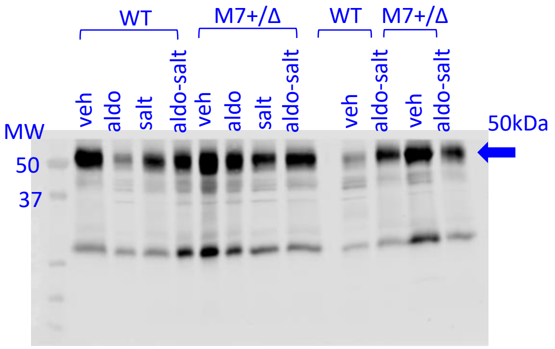

Supplementary Figure 22

Figure 5d – Hearts – PTEN

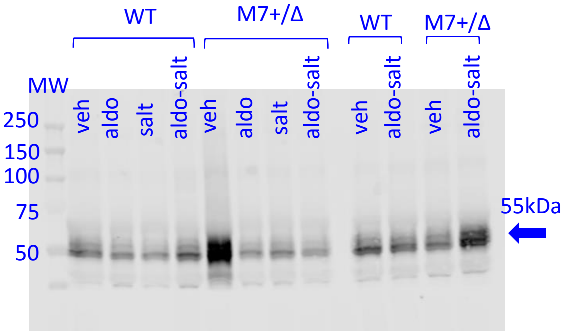

Figure 5d – Hearts – α-tubulin

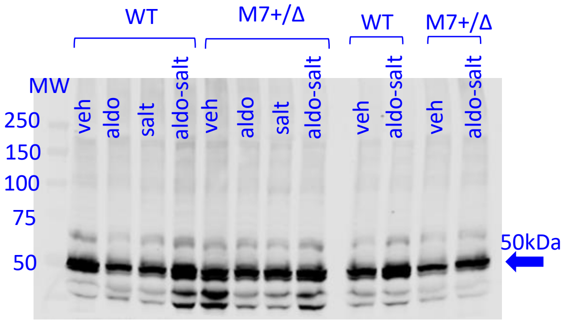

Figure 5e – Kidneys – PTEN

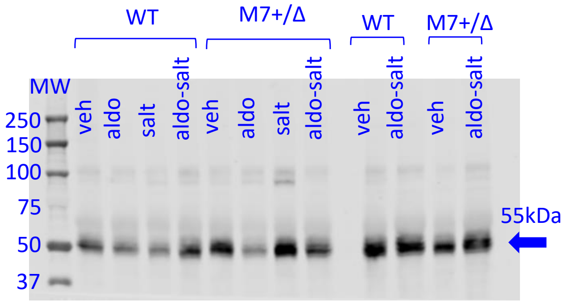

Figure 5e – Kidneys – α-tubulin

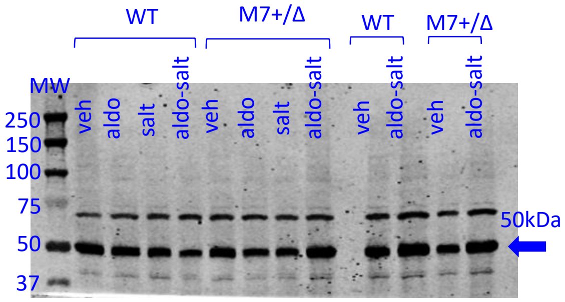

Supplementary Figure 23

Figure 6a – p-Smad3

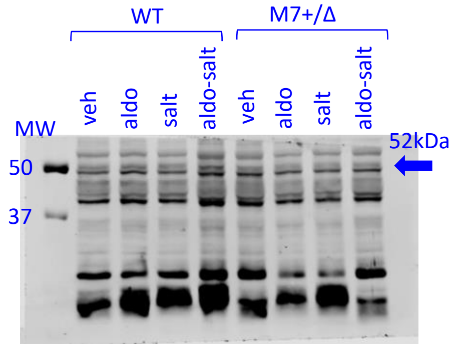

Figure 6a – total-Smad3

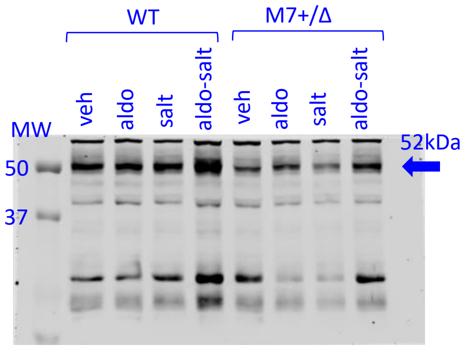

Figure 6a – α-tubulin

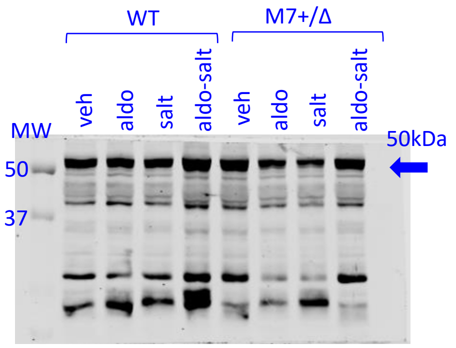

Figure 6b – p-ERK1/2

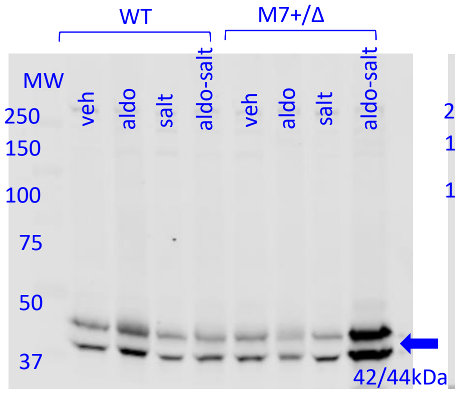

Figure 6b – total-ERK1/2

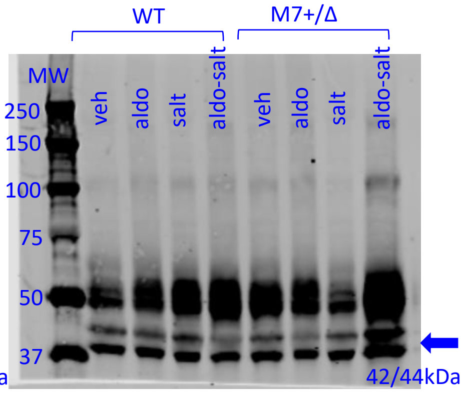

Figure 6b – α-tubulin

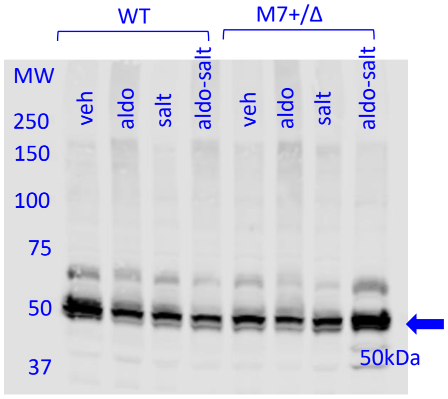

Figure 6c – p-Stat1

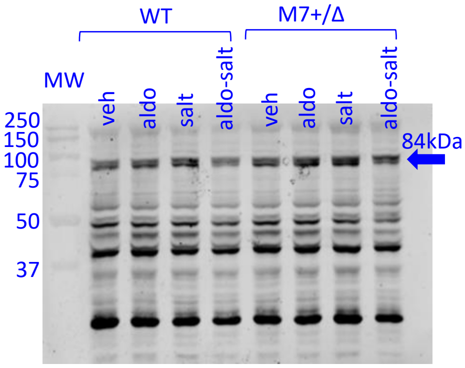

Figure 6c – total-Stat1

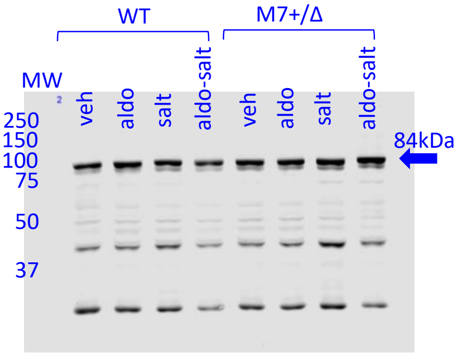

Figure 6c – α-tubulin

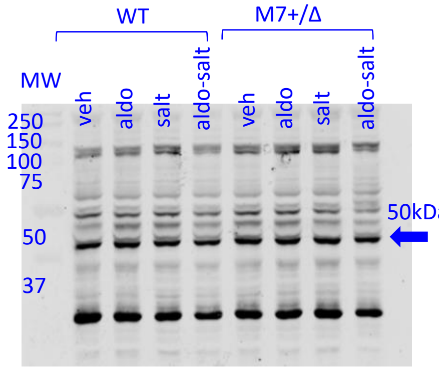

Figure 6d – TGFβ1

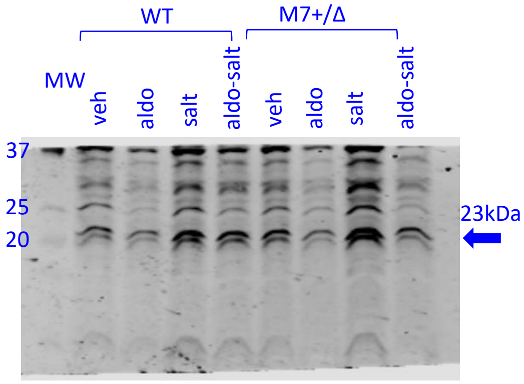

Figure 6d – α-tubulin

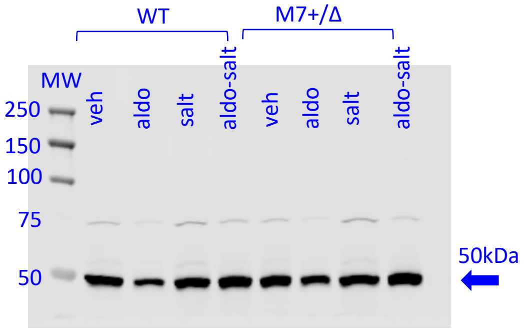

Supplementary Figure 24

Figure 7a – p-ERK1/2

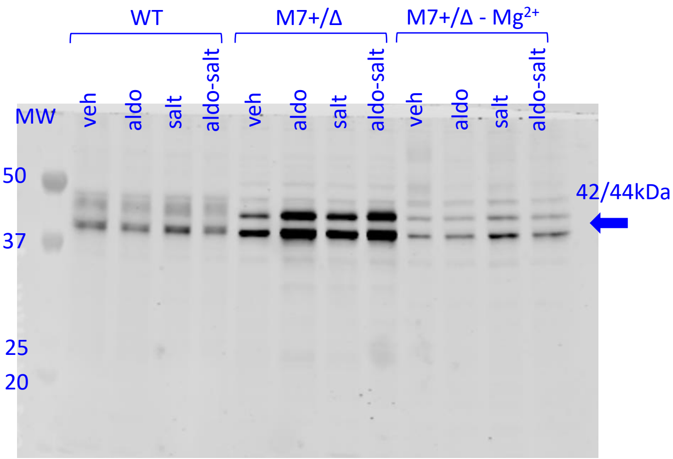

Figure 7a – total-ERK1/2

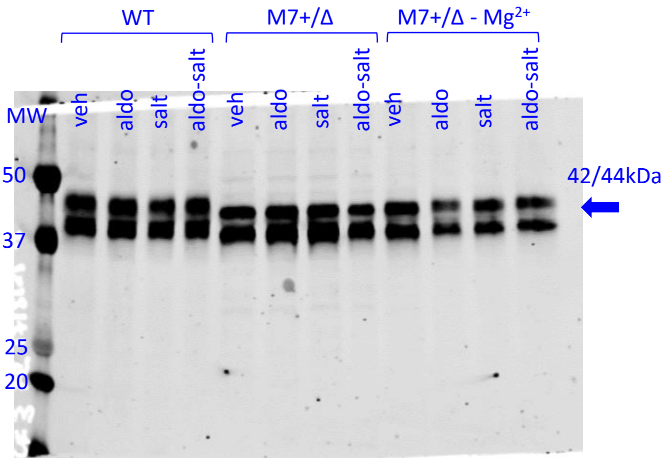

Figure 7b – p-Smad3

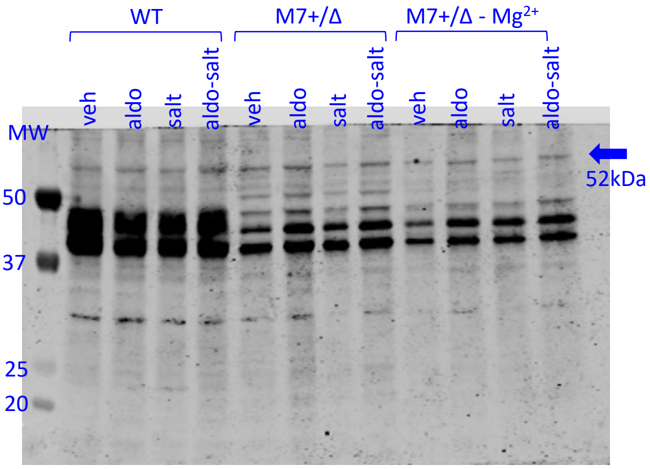

Figure 7b – total-Smad3

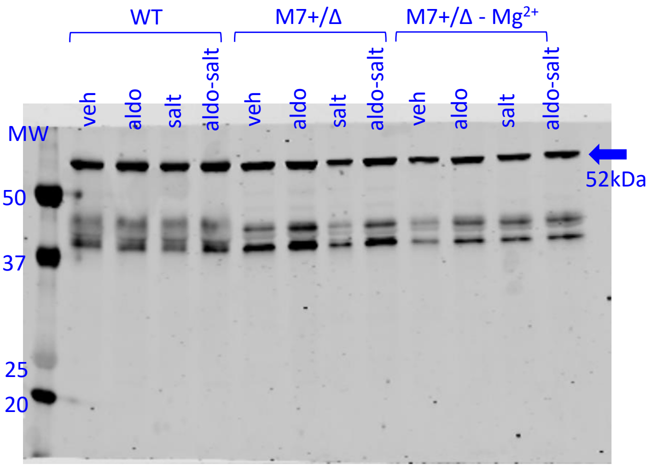

Supplementary Figure 25

Figure 7c – p-Stat1

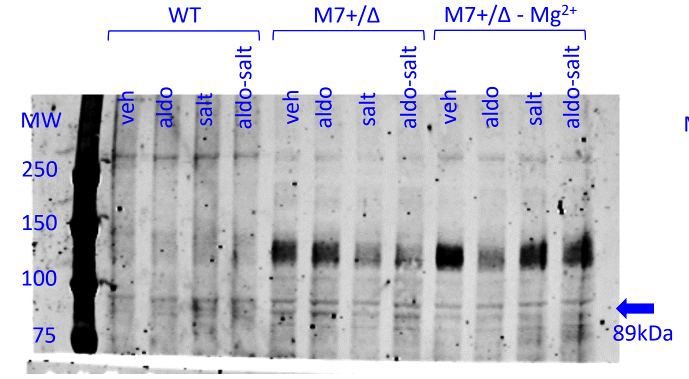

Figure 7c – total-Stat1

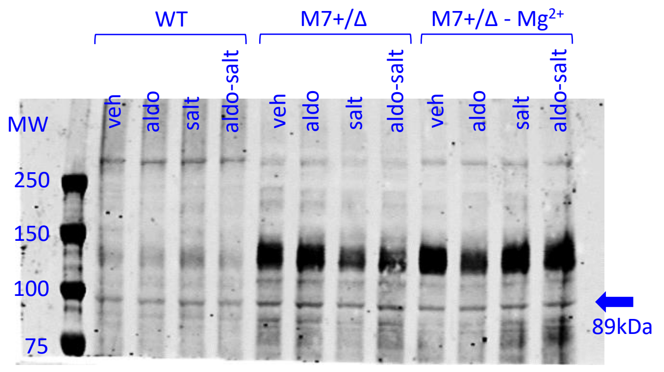

Figure 7d – p-Stat1

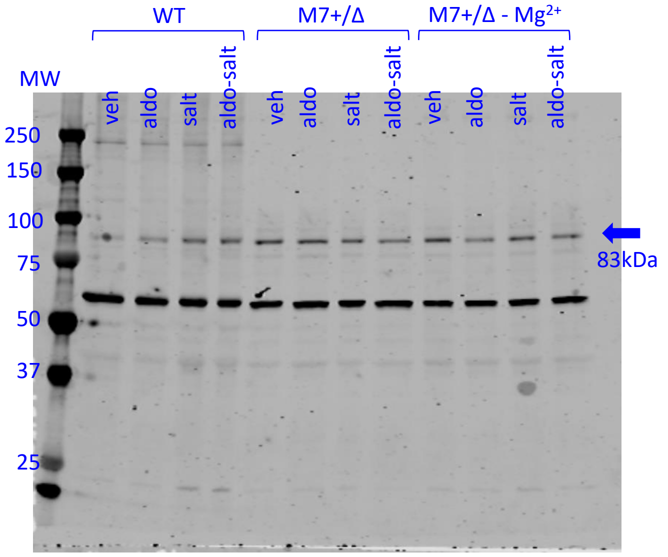

Figure 7d – total-Stat1

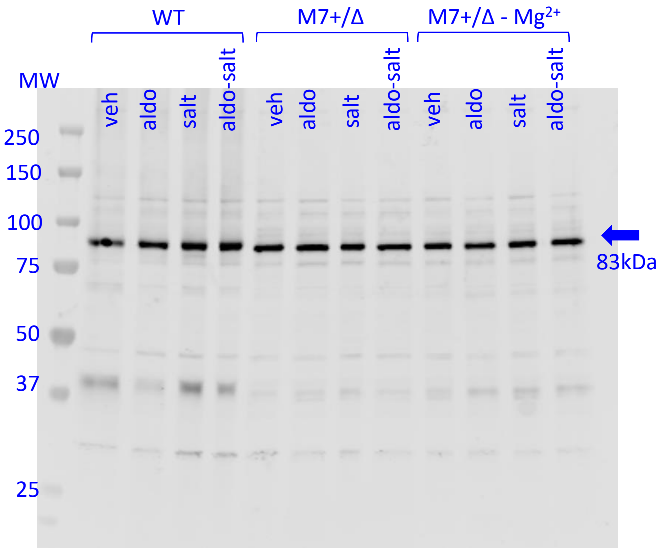

Supplement: Supplementary file 2 — Supplementary Information [file 42003_2022_3715_MOESM2_ESM.pdf]
