# Supplementary material for: TRPM7 deficiency exacerbates cardiovascular and renal damage induced by aldosterone-salt
Source: Commun Biol. 2022 Jul 26;5:746. doi: 10.1038/s42003-022-03715-z (PMC9325869; doi:10.1038/s42003-022-03715-z)
Supplement: Supplementary file 3 — Description of Additional Supplementary Files [file 42003_2022_3715_MOESM3_ESM.pdf]

# Description of Additional Supplementary Files

**File name:** Supplementary Data
**Description:** All data underlying the graphs and charts presented in the main figures.
