# Supplementary material for: TRPM7 deficiency exacerbates cardiovascular and renal damage induced by aldosterone-salt
Source: Commun Biol. 2022 Jul 26;5:746. doi: 10.1038/s42003-022-03715-z (PMC9325869; doi:10.1038/s42003-022-03715-z)
Supplement: Supplementary file 5 — Reporting Summary [file 42003_2022_3715_MOESM5_ESM.pdf]

nature research

Corresponding author(s): Rhian M. Touyz
Francisco J. Rios

Last updated by author(s): Jul 4, 2022

Reporting Summary

Nature Research wishes to improve the reproducibility of the work that we publish. This form provides structure for consistency and transparency in reporting. For further information on Nature Research policies, see our Editorial Policies and the Editorial Policy Checklist.

Statistics

For all statistical analyses, confirm that the following items are present in the figure legend, table legend, main text, or Methods section.

n/a	Confirmed	
☐	☒	The exact sample size (*n*) for each experimental group/condition, given as a discrete number and unit of measurement
☐	☒	A statement on whether measurements were taken from distinct samples or whether the same sample was measured repeatedly
☐	☒	The statistical test(s) used AND whether they are one- or two-sided<br>*Only common tests should be described solely by name; describe more complex techniques in the Methods section.*
☒	☐	A description of all covariates tested
☐	☒	A description of any assumptions or corrections, such as tests of normality and adjustment for multiple comparisons
☐	☒	A full description of the statistical parameters including central tendency (e.g. means) or other basic estimates (e.g. regression coefficient) AND variation (e.g. standard deviation) or associated estimates of uncertainty (e.g. confidence intervals)
☐	☒	For null hypothesis testing, the test statistic (e.g. *F*, *t*, *r*) with confidence intervals, effect sizes, degrees of freedom and *P* value noted<br>*Give P values as exact values whenever suitable.*
☒	☐	For Bayesian analysis, information on the choice of priors and Markov chain Monte Carlo settings
☒	☐	For hierarchical and complex designs, identification of the appropriate level for tests and full reporting of outcomes
☒	☐	Estimates of effect sizes (e.g. Cohen's *d*, Pearson's *r*), indicating how they were calculated

Our web collection on statistics for biologists contains articles on many of the points above.

Software and code

Policy information about availability of computer code

Data collection	Flow cytometry - BD FACSDivaTM software version 6.1.3 - BD Biosciences<br>Blood pressure - BP-2000 Blood Pressure Analysis System<br>Real time PCR - QuantStudio™ 12K Flex Software version 1.3<br>Chemiluminescence for ROS production - Simplicity version 4.20 4.2.0.0 (2006) for windows.<br>western blot images - Image Studio version 502<br>Microscopy Image acquisition - EVOS xl core version 1.0.198<br>Electrolytes - ISE indirect for Cobas. Roche Cobas C311 autoanalyzer. Roche Diagnostics GmbH.<br>Wire myography - LabChart Pro, DMT is a life science company.<br>Pressure Myography - MyoVIEW software (Danish Myo Technology A/S, version 2.0)
Data analysis	FlowJo software version 7.2.4 (TreeStar, Ashland, USA)<br>Image Studio™ Lite free version (LICOR, Cambridge, UK).<br>Fiji Image J (https://imagej.net/Fiji)<br>Graphpad Prism for windows (version 6.0.1)<br>Microsoft excel - Microsoft Office 365<br>Wire myography - LabChart free edition

For manuscripts utilizing custom algorithms or software that are central to the research but not yet described in published literature, software must be made available to editors and reviewers. We strongly encourage code deposition in a community repository (e.g. GitHub). See the Nature Research guidelines for submitting code & software for further information.

Data

Policy information about availability of data

All manuscripts must include a data availability statement. This statement should provide the following information, where applicable:

- Accession codes, unique identifiers, or web links for publicly available datasets
- A list of figures that have associated raw data
- A description of any restrictions on data availability

The datasets generated during and/or analysed during the current study are available from the corresponding author on reasonable request.

Field-specific reporting

Please select the one below that is the best fit for your research. If you are not sure, read the appropriate sections before making your selection.

☒ Life sciences ☐ Behavioural & social sciences ☐ Ecological, evolutionary & environmental sciences

For a reference copy of the document with all sections, see nature.com/documents/nr-reporting-summary-flat.pdf

Life sciences study design

All studies must disclose on these points even when the disclosure is negative.

Sample size	Sample size for mouse experiments were determined using a standard power calculation based on estimated effect size.
Data exclusions	western blot and PCR data were excluded based on sample degradation, as observed by the expression of the housekeeping protein or gene. No other samples were excluded.
Replication	Experiments were repeated multiple times and n numbers are included to indicate the number of replicates per panel.
Randomization	Littermate mice from each genotype were randomized into different groups groups based on random caging at weaning.
Blinding	Blinding experiments were performed for myography, histology, Flow cytometry, and electrolytes measurements.

Reporting for specific materials, systems and methods

We require information from authors about some types of materials, experimental systems and methods used in many studies. Here, indicate whether each material, system or method listed is relevant to your study. If you are not sure if a list item applies to your research, read the appropriate section before selecting a response.

Materials & experimental systems

n/a	Involved in the study	
☐	☒	Antibodies
☒	☐	Eukaryotic cell lines
☒	☐	Palaeontology and archaeology
☐	☒	Animals and other organisms
☒	☐	Human research participants
☒	☐	Clinical data
☒	☐	Dual use research of concern

Methods

n/a	Involved in the study	
☒	☐	ChIP-seq
☐	☒	Flow cytometry
☒	☐	MRI-based neuroimaging

Antibodies

Antibodies used

Antibodies used for Western blot:
- Beta-actin, Sigma Aldrich, mouse monoclonal, clone AC-74, cat# A5316, 1:5000
- Phospho-Smad3 (S423 + S425), rabbit monoclonal, Abcam, clone EP823Y, cat# ab52903, 1:500
- Total-Smad3, mouse monoclonal, Santa Cruz Biotechnology, clone 38-Q, cat# sc-101154, 1:1000
- Peroxiredoxin-SO3, rabbit polyclonal, Abcam, cat# ab16830, 1:1000
- Alpha-Tubulin, rabbit polyclonal, Abcam, cat# ab176560, 1:5000
- IL-6, rabbit monoclonal, Abcam, clone EPR21711, cat# ab233706, 1:1000
- TGFβ1, rabbit polyclonal, Abcam, cat# ab92486, 1:1000
- IL-11, mouse monoclonal, Biolegend, clone M3103F11, cat# 677602, 1:1000
- Phospho-Stat3 (Tyr705), mouse monoclonal, Cell Signaling, Clone 3E2, cat# 9138S, 1:1000
- Total-Stat3, rabbit monoclonal, Cell Singanling, clone 79D7, cat# 4904S, 1:1000
- Phospho-Stat1 (Tyr701), mouse monoclonal, Abcam, clone M135, cat# ab29045, 1:1000
- Phospho-Stat1 (Tyr701), rabbit monoclonal, Cell Signaling, clone 58D6, cat# 9167S, 1:1000

- Total-Stat1, rabbit monoclonal, Abcam, clone EPR21057-141, cat# ab234400, 1:1000
- Total-Stat1, mouse monoclonal, Cell Signaling, clone 9H2, cat# 9176S, 1:1000
- Phospho-ERK1/2 (Thr202/Tyr204), rabbit monoclonal, Cell Signaling, clone D13.14.4E, cat# 4370, 1:1000
- Total-ERK1/2, mouse monoclonal, Santa Cruz Biotechnology, clone C-9, cat# sc-514302, 1:1000
- PTEN, mouse monoclonal, Santa Cruz Biotechnology, clone A2B1, cat# sc7974, 1:1000
- PPM1A, mouse monoclonal, RnD Systems, clone 498922, cat# MAB4150, 1:1000
- Oxidized PTP, mouse monoclonal, RnD Systems, clone 335636 , cat# MAB2844, 1:1000
- Nox4, rabbit monoclonal, Abcam, clone UOTR1B492, cat# ab109225, 1:1000
- Nox1, goat polyclonal, Sigma-Aldrich, cat# SAB2501686, 1:1000
- Phospho-TRPM7 (Ser1511), rabbit polyclonal, kindly gifted by Dr Chubanov, University of Munich, Germany, 1:1000
- TRPM7, mouse monoclonal, Thermo Fisher, clone S74-25, cat# MA5-27620, 1:1000
- alphaENaC, mouse monoclonal, Abcam, cat# ab214192, 1:1000
- Na+/K+ ATPase 1, ATP1A1 rabbit Polyclonal antibody, Proteintech, cat# 14418-1-AP, 1:1000.

Antibodies used for Flow Cytometry
- anti-mouse CD45-FITC, Biolegend, clone 30-F11, cat#103108, 1:600
- anti-mouse CD3-PE-Cy7, Biolegend, clone 145-2C11, cat# 100320, 1:50
- anti-mouse CD4-APC, Biolegend, clone GK1.5, cat# 100412, 1:300
- anti-mouse CD8-APC-Cy7, Biolegend, clone, 53-6.7, cat# 100714, 1:300
- anti-mouse F4/80-Alexa-647, Biolegend, clone BM8, cat# 123122, 1:300
- anti-mouse CD11c-PE-Cy7, Biolegend, clone N418, cat# 117318, 1:300
- anti-mouse CD206-FITC, Biolegend, clone C068C2, cat# 141704, 1:300
- anti-mouse CD45-PE, BD Pharmingen, clone 30-F11, cat# 553081, 1:300

Validation

Antibodies from commercial vendors were tested on known positive samples. Antibody from our collaborators were validated in previous publications.

Animals and other organisms

Policy information about studies involving animals; ARRIVE guidelines recommended for reporting animal research

Laboratory animals

Mice background: Wild type (WT) mice (C57BL/6J and SV129 mixed background) and mice heterozygous for the deletion of the TRPM7-kinase (TRPM7+/Δkinase), generated by the gene-targeting vector technique.
We used only male mice and experiments started when they were 12-16 weeks of age.

Wild animals

N/A

Field-collected samples

N/A

Ethics oversight

Animal experiments were approved by the University of Glasgow Animal Welfare and Ethics Review Board in accordance with the United Kingdom Animals Scientific Procedures Act 1986 (Licence No. 70/9021) and with ARRIVE Guidelines.

Note that full information on the approval of the study protocol must also be provided in the manuscript.

Flow Cytometry

Plots

Confirm that:

☒ The axis labels state the marker and fluorochrome used (e.g. CD4-FITC).

☒ The axis scales are clearly visible. Include numbers along axes only for bottom left plot of group (a 'group' is an analysis of identical markers).

☒ All plots are contour plots with outliers or pseudocolor plots.

☒ A numerical value for number of cells or percentage (with statistics) is provided.

Methodology

Sample preparation

Kidneys were cut in small pieces ≤ 1 mm and digested in collagenase II (2 mg/mL) (Sigma-Aldrich, Dorset, UK) diluted in DMEM serum free with constant agitation at 37 C for 40 min. Enzyme was inactivated by adding FBS 10% in final concentration. Digested tissues were filtered through a 70 µm cell strainer.
Spleen cells were collected after mechanic disruption and filtered through a 70 µm cell strainer. Cell suspension obtained from kidneys and spleens were centrifuged at 300 x g for 10 min.
The cell pellet obtained from kidneys and spleens was resuspended in ACK Lysis Buffer (NH4Cl 150 mmol/L, KHCO3 10 mmol/L, Na2EDTA 0.1 mmol/L, pH 7.2–7.4) for 3 min on ice for erythrocytes disruption, followed by addition of 40 mL of PBS/FBS 2% and centrifugation at 300 x g for 10 min. Total cell number was obtained by counting in a Neubauer chamber, using trypan blue 0.4% exclusion. Cells were resuspended in FACS buffer (PBS/FBS 2%, NaN3 0.05%) followed by staining with specific antibodies.

Instrument

FACS Canto II flow cytometer (BD Biosciences, Wokingham, UK)

Software	Data acquisition was performed using the software BD FACSDivaTM version 6.1.3 - BD Biosciences<br>Data analysis was performed using FlowJo software version 7.2.4 (TreeStar, Ashland, USA)
Cell population abundance	No cell sorting was performed. We performed flow cytometry to see different cell population.
Gating strategy	ll flow cytometry experiments used preliminary gates for: FSC singlets, SSC singlets, cell population of interest by FSC and SSC.<br>Hematopoietic cells were identified as CD45+. Cell identification was performed as follow:<br>- Total macrophages: CD45+F4/80+ cells; M1 macrophages: F4/80+ CD11+ CD206-; M2 macrophages F4/80+ CD11c- CD206+<br>- Total lymphocytes: CD45+ CD3+; CD4 T cells: CD3+ CD4+ CD8-; CD8 T cells: CD3+ CD4- CD8+

☐ Tick this box to confirm that a figure exemplifying the gating strategy is provided in the Supplementary Information.
